# Density-Functionalized QM/MM Delivers Chemical Accuracy For Solvated Systems

**DOI:** 10.1021/acs.jctc.5c01440

**Published:** 2025-10-15

**Authors:** Xin Chen, Jessica A. Martinez B, Xuecheng Shao, Marc Riera Riambau, Oliviero Andreussi, Francesco Paesani, Michele Pavanello

**Affiliations:** † Department of Physics, 242613 Rutgers University, Newark, New Jersey 07102, United States; ‡ Department of Chemistry, 67206 Rutgers University, Newark, New Jersey 07102, United States; § Key Laboratory of Material Simulation Methods and Software of Ministry of Education, College of Physics, Jilin University, Changchun 130012, China; ∥ Department of Chemistry, 8784 University of California-San Diego, San Diego, California 92093, United States; ⊥ Department of Chemistry, 1791 Boise State University, Boise, Idaho 83725, United States

## Abstract

We present a reformulation of QM/MM as a fully quantum mechanical theory of interacting subsystems, all treated at the level of density functional theory (DFT). For the MM subsystem, which lacks orbitals, we assign an ad hoc electron density and apply orbital-free DFT functionals to describe its quantum properties. The interaction between the QM and MM subsystems is also treated using orbital-free density functionals, accounting for Coulomb interactions, exchange, correlation, and Pauli repulsion. Consistency across QM and MM subsystems is ensured by employing data-driven, many-body MM force fields that faithfully represent DFT functionals. Applications to water-solvated systems demonstrate that this approach achieves unprecedented, very rapid convergence to chemical accuracy as the size of the QM subsystem increases. We validate the method with several pilot studies, including water bulk, water clusters (prism hexamer and pentamers), solvated glucose, a palladium aqua ion, and a wet monolayer of MoS_2_.

## Graphical abstract

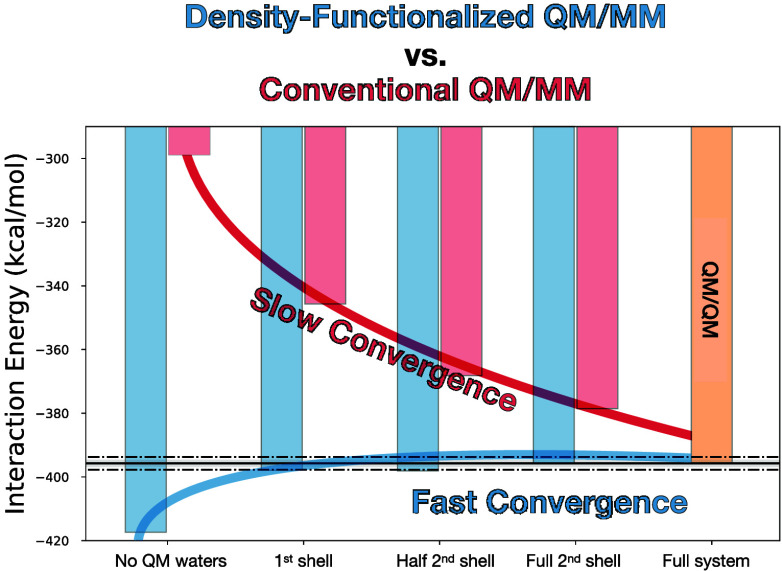

## 1 Introduction

QM/MM (standing for quantum mechanics/molecular mechanics) has revolutionized computational biochemistry.^1^ Since the pioneering work of Honig and Karplus,^2^ the combination of a quantum mechanical (QM) description for a subsystem with a classical point-charge description of its environment has led to major breakthroughs in fields such as enzymatics,^3,4^ drug development,^5,6^ and materials design.^7^ Since its conception, methods handling the QM and MM subsystems have dramatically evolved. Today’s MM force fields can integrate data-driven potentials,^8^ polarizable models,^9–15^ and even machine learning techniques.^16,17^ QM methods have also evolved dramatically, ranging from DFT to wave function theory methods routinely used in conjunction with QM/MM.^18^

The nature of the QM-MM interaction has also evolved. Initially, these were handled mechanically,^3^ with Coulomb interactions calculated *a posteriori*, influencing only the forces and total energy, but without affecting the QM wave function or density. The advent of electrostatic embedding improved accuracy by incorporating MM partial charges directly into the QM Hamiltonian.^19^ Ultimately, mutual QM-MM polarization was achieved using polarizable force fields,^9,13^ a concept anticipated in early QM/MM work.^20^

The computational cost of QM/MM simulations is greatly reduced compared to fully quantum mechanical treatments. However, incorporating QM/polarizable-MM interactions in an efficient manner remains challenging. Methods based on judicious partitioning of the induction response have demonstrated excellent scalability.^14,21^ Additionally, algorithmic advances have been supported by steady progress in software development.^17,22–28^ To extend QM/MM simulations to condensed phases, periodic boundary conditions (PBC) have been implemented. Ewald summation techniques are commonly used, and adaptations for molecular condensed phases^29–32^ and material systems^26,28,33,34^ are now widely available.

How accurate are QM/MM models? A common way to address this question is to evaluate the convergence of the results with respect to the size of the QM subsystem or to simply compare against full QM calculations^35^ for selected model systems. Protein environments, for example, are exceptionally complex, and the search for effective ways to include relevant protein regions in QM/MM simulations continues.^36–39^ Particularly challenging has been capturing charge transfer interactions on larger scales,^4,38^ or finding appropriate cluster model systems of enzymatic active sites.^41^

A slow convergence of the QM/MM setup has also affected those systems where partitioning in QM and MM subsystems involves no bond breaking. For example, water solvation.^42^ Ironically, independent QM-only or MM-only treatments of liquid water can provide accurate results, but their combination in QM/MM workflows results in an overall reduced accuracy.^26,43^ Accurately modeling aqueous environments with QM/MM is essential due to the need to consider large water environments to properly account for the static and dynamic responses at water-material interfaces.^44,45^ Therefore, representing these polarization effects in water bulk, which can extend for several nanometers, is crucial for capturing significant effects on the energetics of solvated species.^46,47^

The culprit is the difficulty to accurately capture QM-MM interactions with a computationally efficient method. The MM subsystem is typically described using methods that largely (or completely) neglect its electronic structure. Point charges or, at times, point polarizable dipoles do not faithfully represent any electronic structure! The polarizable density embedding method^48^ tackles this problem by dividing the MM subsystem into two regions: one near the QM subsystem is assigned a QM density derived from isolated fragment calculations, while the remaining MM atoms are treated using conventional point charge or dipole models. This embedding approach improves the accuracy of the QM Hamiltonian by capturing both electrostatic and nonelectrostatic interactions, leading to better results than traditional QM/MM setups.^49^ Several related approaches build on the above ideas, such as QM/ESP,^50^ QM/GEM,^51^ QXD,^52^ density embedding methods,^53^ and many-body expansions.^54^ There have also been efforts to include the quantum mechanical Pauli repulsion in QM/MM, but with mixed success.^55^ Some strategies address this issue by parametrizing the mechanical embedding interaction energy without adding new terms to the QM Hamiltonian,^56–58^ while others also include the effects of Pauli repulsion on the QM system by placing pseudopotentials in the MM region.^59^

Thus, our approach in this work is to treat QM and MM subsystems on a more similar footing, aiming to reduce the impact of an imbalanced QM-MM interface and an imbalanced treatment of the internal energy of QM and MM subsystems. We propose “density-functionalizing” the MM subsystem, assigning it an electron density such that it can be handled like an electronic subsystem within the rigorous framework of subsystem DFT (sDFT).^60–64^ This standardization of QM and MM subsystems allows for the use of first-principles density functionals for evaluating the QM-MM interaction, inherently capturing all relevant physical effects, such as exchange, correlation, Pauli repulsion, electrostatics, and charge penetration. Accounting for Pauli repulsion is a crucial aspect of sDFT. This is achieved through nonadditive functionals as demonstrated early on in the subsystem DFT literature.^65^ In particular, the nonadditive kinetic energy functional formally encodes Pauli repulsion and prevents charge “spill-out” across subsystems.^66,67^ The next section details the theoretical framework for the density-functionalized QM/MM approach, with additional and less critical details provided in the Supporting Information.^68^

## 2 Density-Functionalization of QM/MM

The central idea is to assign an electron density to both the QM subsystem, ρ_QM_(**r**), and the MM subsystem, ρ_MM_(**r**), with the total electron density given by their sum and the energy functional borrowed from rigorous sDFT^60–62^

1 $$\rho \left(r\right)=\rho_{\mathrm{QM}}\left(r\right)+\rho_{\mathrm{MM}}\left(r\right)$$

2 $$E\left[\rho_{\mathrm{QM}},\rho_{\mathrm{MM}}\right]=E\left[\rho_{\mathrm{QM}}\right]+E\left[\rho_{\mathrm{MM}}\right]+E^{\mathrm{nad}}\left[\rho_{\mathrm{QM}},\rho_{\mathrm{MM}}\right]$$

Although formally the electronic energy is strictly a density functional, in practice, the external potential (electron–nuclear attraction) is known ahead of time and is thus specified for the QM subsystem, *v*_QM_(**r**), and for the MM subsystem, *v*_MM_(**r**), such that the additive part of the energy is given by (disregarding for the time being the nuclear–nuclear repulsion)

3 $$E\left[\rho_{\mathrm{QM}}\right]=T_{s}\left[\rho_{\mathrm{QM}}\right]+E_{H}\left[\rho_{\mathrm{QM}}\right]+E_{\mathrm{xc}}\left[\rho_{\mathrm{QM}}\right]+\int v_{\mathrm{QM}}\left(r\right)\rho_{\mathrm{QM}}\left(r\right)dr$$

4 $$E\left[\rho_{\mathrm{MM}}\right]=T_{s}\left[\rho_{\mathrm{MM}}\right]+E_{H}\left[\rho_{\mathrm{MM}}\right]+E_{\mathrm{xc}}\left[\rho_{\mathrm{MM}}\right]+\int v_{\mathrm{MM}}\left(r\right)\rho_{\mathrm{MM}}\left(r\right)dr$$

where *T*_s_, *E*_H_ and *E*_xc_ are the noninteracting kinetic energy, the classical electron–electron repulsion (Hartree) and the exchange-correlation functionals, respectively. The nonadditive energy is thus given by

5 $$E^{\mathrm{nad}}\left[\rho_{\mathrm{QM}},\rho_{\mathrm{MM}}\right]=T_{s}^{\mathrm{nad}}\left[\rho_{\mathrm{QM}},\rho_{\mathrm{MM}}\right]+E_{\mathrm{xc}}^{\mathrm{nad}}\left[\rho_{\mathrm{QM}},\rho_{\mathrm{MM}}\right]+\int \frac{\rho_{\mathrm{QM}}\left(r\right)\rho_{\mathrm{MM}}\left(r\right)}{\left|r-r^{\prime }\right|}drdr^{\prime }+\int \rho_{\mathrm{MM}}\left(r\right)v_{\mathrm{QM}}\left(r\right)dr+\int \rho_{\mathrm{QM}}\left(r\right)v_{\mathrm{MM}}\left(r\right)dr$$

where *T*_s_^nad^[ρ_QM_, ρ_MM_] = *T*_s_[ρ_QM_ + ρ_MM_] – *T*_s_[ρ_QM_] – *T*_s_[ρ_MM_] and equivalently for *E*_xc_^nad^.

The equations above can be exploited for a variational minimization of the energy functional with respect to variations in both ρ_QM_ and ρ_MM_, provided that suitable approximations for the relevant density functionals are available.

When both QM and MM subsystems are described at the Kohn–Sham DFT level, the resulting sDFT framework is highly accurate, especially in regimes of weak intersubsystem interactions. Notably, sDFT with semilocal functionals for *E* ^nad^ has consistently demonstrated excellent agreement with experimental structure factors of condensed-phase systems, such as liquid water,^69^ fluid CO_2_,^70^ and solvated ionic species.^71^ Sub-1 kcal/mol accuracy is now routinely attained in sDFT calculations, whether using Kohn–Sham subsystems,^72–74^ orbital-free subsystems,^75^ or advanced multiscale schemes.^71,76–78^ The treatment of hydrogen-bonding interactions, which are central to this work, is particularly reliable when employing the nonadditive PBE exchange-correlation in conjunction with the revAPBEk nonadditive kinetic energy,^72^ as well as other GGA nonadditive functionals.^79^ Thus, we have confidence that our approach can accurately capture QM-MM interactions for solvated species.

In a QM/MM framework, eq 4 is typically replaced by a classical force field expression. This classical term includes electrostatic interactions between atom-centered point charges and, in some cases, atom-centered polarizable dipoles, as well as empirical expressions for short-range dispersion-repulsion interactions and bonded terms. In most QM/MM implementations, the classical component is handled by a separate tool or software package responsible for evaluating this energy contribution. However, the electrostatic multipoles defined in the MM force field also contribute to the electrostatic terms in the QM-MM interaction energy, as given in eq 5. To account for short-range dispersion-repulsion interactions and to prevent unphysical behavior when the QM and MM subsystems are close, ad hoc corrections are commonly introduced to replace the first two terms of eq 5. Once the dependence of eq 2 on the atomic positions of the MM subsystem is established, taking the functional derivative of eq 2 with respect to ρ_QM_(**r**) enables the optimization of the QM subsystem using a self-consistent field (SCF) approach.

The point charges and point dipoles used to represent the MM subsystem can be parametrized in different ways. They may be adjusted to fit empirical data for the MM system, or optimized to reproduce properties related to the electronic density of the MM components as computed from first-principles, such as binding energies or electrostatic potentials. Since the permanent charges in most MM force fields do not depend on the molecular geometry or environment, atomic polarizabilities are often introduced into the force field to capture the system’s electrostatic response to external fields. In these polarizable force fields, the nonadditive term in eq 5 induces dipoles in the MM subsystem, leading to a situation where the QM and MM subsystems mutually polarize each other.

The standard approximations in conventional QM/MM approaches often result in notable inaccuracies, especially when the QM/MM boundary is not fixed but systematically changes. Motivated by the progress of methods that incorporate explicit electron densities for the MM subsystem,^48,80^ as well as the robust performance of the sDFT framework, we propose that a density-functional-based QM/MM approach grounded in eqs 2–5 can yield highly accurate models of solute–solvent systems. In particular, this framework is expected to be reliable for systems where the QM and MM subsystems interact weakly, provided that well-defined and consistent mappings exist between first-principles electronic densities and the corresponding force-field multipoles. Establishing such mappings enables a seamless and physically consistent description of both forward (from electronic density to multipoles) and backward (from multipoles to electronic density) interactions, which is essential for capturing mutual polarization and accurate QM-MM coupling in complex environments.

For the forward mapping from electronic-structure calculations to classical electrostatics, the many body polarizable approach developed by Paesani and collaborators demonstrates that it is possible to accurately describe statistical properties of bulk systems by carefully parametrizing a classical force field using a large database of high-level few-body first-principles simulations.^81,82^ The early work on MB-pol focused on coupled cluster calculations for water dimers and trimers, but the same methodology can be applied to DFT-based calculations, as in the case of MB-PBE.^83^ The resulting force fields describe electrostatic interactions in terms of permanent charges and polarizabilities. As in similar approaches, short-range corrections are included to prevent self-polarization and to avoid the unphysical divergence of induced dipoles when polarizable sites are very close. In this work, we propose that using an MM subsystem with electrostatic interactions that are fully consistent with the quantum mechanical treatment of the QM region will allow for accurate and seamless convergence of QM/MM calculations as the boundary between the two regions changes.

For the backward mapping, which goes from the classical force field in the MM region to an effective electronic density used in the density functional QM/MM interaction, we adopt the following strategy. We represent the atomic nuclei (of QM and MM subsystems) with pseudopotentials. To keep calculations efficient for large MM subsystems, we use the local part of the ultrasoft pseudopotentials introduced by Garrity and Vanderbilt (GBRV),^84^ which are designed for simulations that require low plane wave cutoffs and high throughput. This assignment is carried out efficiently using the particle mesh Ewald method, which scales linearly with system size.^85^ For the valence electrons, we convert the point multipoles calculated by the classical force field into a smooth electronic charge density.

For each MM site *i*, we represent the valence electron density by a Gaussian function centered at the site position, **R**_*i*_, with an adjustable width σ_*i*_. Specifically, the density is given by ρ_*q*_*i*__(**r**) = (*N*_*i*_ – *q*_*i*_) *g*_σ_*i*__(**r** – **R**_*i*_), where *g*_σ_*i*__ is a normalized Gaussian. The prefactor ensures an accurate description of the ion’s permanent charge *q*_*i*_, while correctly accounting for the total number of valence electrons *N*_*i*_ associated with the isolated atom. If the force field includes higher multipoles in its electrostatic interactions, we incorporate these into the reconstructed electron density by using derivatives of the normalized Gaussian. For example, a point dipole can be mapped onto a smooth charge density by taking the gradient of a Gaussian, $\rho_{\mu_{j}}\left(r\right)=-\vec{\mu_{j}}\cdot \mathrm{\nabla ⃗}g_{\sigma_{j}}\left(r-R_{j}\right)$ , where $\vec{\mu_{j}}$ is the induced dipole at site *j*. Higher-order multipoles can be included in an analogous manner using higher derivatives. The total valence electron density is then given by the sum of these contributions

6 $$\rho_{\mathrm{MM}}\left(r\right)=\sum_{i\in \mathrm{MM}\;\mathrm{charges}}\rho_{q_{i}}\left(r\right)+\sum_{j\in \mathrm{MM}\;\mathrm{dipoles}}\rho_{\mu_{j}}\left(r\right)$$

We stress that the proposed formulation relies on a set of element-specific widths (the σ_*i*/*j*_) that can significantly affect the final shape of the reconstructed electronic density (both permanent charges and polarization density due to the induced dipoles).

For the forward mapping, we hypothesize that selecting the Gaussian widths to best reproduce the interaction energies between QM/MM and QM/QM (sDFT) calculations will enable an accurate description of mutual polarization effects. This approach should also ensure that QM/MM calculations converge smoothly as the size of the QM subsystem increases.

The functional derivatives of the nonadditive interaction energy with respect to the QM or MM subsystem densities yields the embedding potentials

7 $$v_{\mathrm{emb}}^{\mathrm{QM}}\left(r\right)=\frac{\delta E^{\mathrm{nad}}\left[\rho_{\mathrm{QM}},\rho_{\mathrm{MM}}\right]}{\delta \rho_{\mathrm{QM}}\left(r\right)}=v_{\mathrm{MM}}\left(r\right)+\int dr^{\prime }\frac{\rho_{\mathrm{MM}}\left(r^{\prime }\right)}{\left|r-r^{\prime }\right|}+\frac{\delta T_{s}^{\mathrm{nad}}}{\delta \rho_{\mathrm{QM}}\left(r\right)}+\frac{\delta E_{\mathrm{xc}}^{\mathrm{nad}}}{\delta \rho_{\mathrm{QM}}\left(r\right)}$$

8 $$v_{\mathrm{emb}}^{\mathrm{MM}}\left(r\right)=\frac{\delta E^{\mathrm{nad}}\left[\rho_{\mathrm{QM}},\rho_{\mathrm{MM}}\right]}{\delta \rho_{\mathrm{MM}}\left(r\right)}=v_{\mathrm{QM}}\left(r\right)+\int dr^{\prime }\frac{\rho_{\mathrm{QM}}\left(r^{\prime }\right)}{\left|r-r^{\prime }\right|}+\frac{\delta T_{s}^{\mathrm{nad}}}{\delta \rho_{\mathrm{MM}}\left(r\right)}+\frac{\delta E_{\mathrm{xc}}^{\mathrm{nad}}}{\delta \rho_{\mathrm{MM}}\left(r\right)}$$

that can be used to optimize the QM or MM degrees of freedom (densities). In particular, eq 7 enters the Hamiltonian of the QM subsystem at each SCF step and it is used for the calculation of the ground state QM electronic densities. The sDFT framework, together with its implementation in the eDFTpy software,^86^ make it possible to couple the QM/MM embedding potential to multiple QM subsystems.

eq 8 can be used by the MM engine to account for the influence of the QM region on induced dipoles and interatomic forces. To include the effect on induced dipoles, the gradient of the MM embedding potential is added to the classical electric field that determines the induced dipoles in the force field. Although all terms in eq 8 should ideally contribute to the embedding field that polarizes the MM subsystem, the current treatment of atomic polarizabilities in polarizable force fields is only well-defined for describing long-range polarization. As a result, in our present implementation, we retain only the first two terms of eq 8, which correspond to classical electrostatic interactions, and neglect the contributions from the nonadditive kinetic and exchange-correlation terms. These omitted terms mainly affect short-range interactions, where the classical force field approach is less reliable.

The evaluation of the QM additive energy in [Disp-formula eq3] can utilize the sDFT implementation of eDFTpy with either a single QM subsystem or multiple QM subsystems. When *N*_S_ QM subsystems are involved, the QM electron density is represented as the sum of contributions from each subsystem, and the QM energy is decomposed into additive and nonadditive terms, following the structure of [Disp-formula eq2]. Namely

9 $$\rho_{\mathrm{QM}}\left(r\right)=\sum_{I=1}^{N_{S}}\rho_{I}\left(r\right)$$

10 $$E\left[\rho_{\mathrm{QM}}\right]=\sum_{I=1}^{N_{S}}E\left[\rho_{I}\right]+E^{\mathrm{nad}}\left[\{\rho_{I}\}\right]$$

## 3 Details of the Implementation

We now describe the implementation of the QM/MM algorithm in the eDFTpy software package.^86^ Figure 1 shows the overall workflow for the QM/MM SCF cycle when a MM subsystem is present. The process begins with initial guesses for both the MM and QM electron densities and wave functions. In eDFTpy, the QM calculations are performed using QEpy,^87^ a Python interface to Quantum ESPRESSO 7.2,^88^ while the MM subsystem is treated using a Python interface to MBX.^89^ The initial guess for the QM electron density is typically constructed from a sum of atomic densities provided in the pseudopotential files. For the MM subsystem, we use a valence electron density obtained through the backward mapping procedure described above, ensuring that it is consistent with the permanent charges assigned in the force field, which remain fixed and independent of geometry.

**Figure 1:**
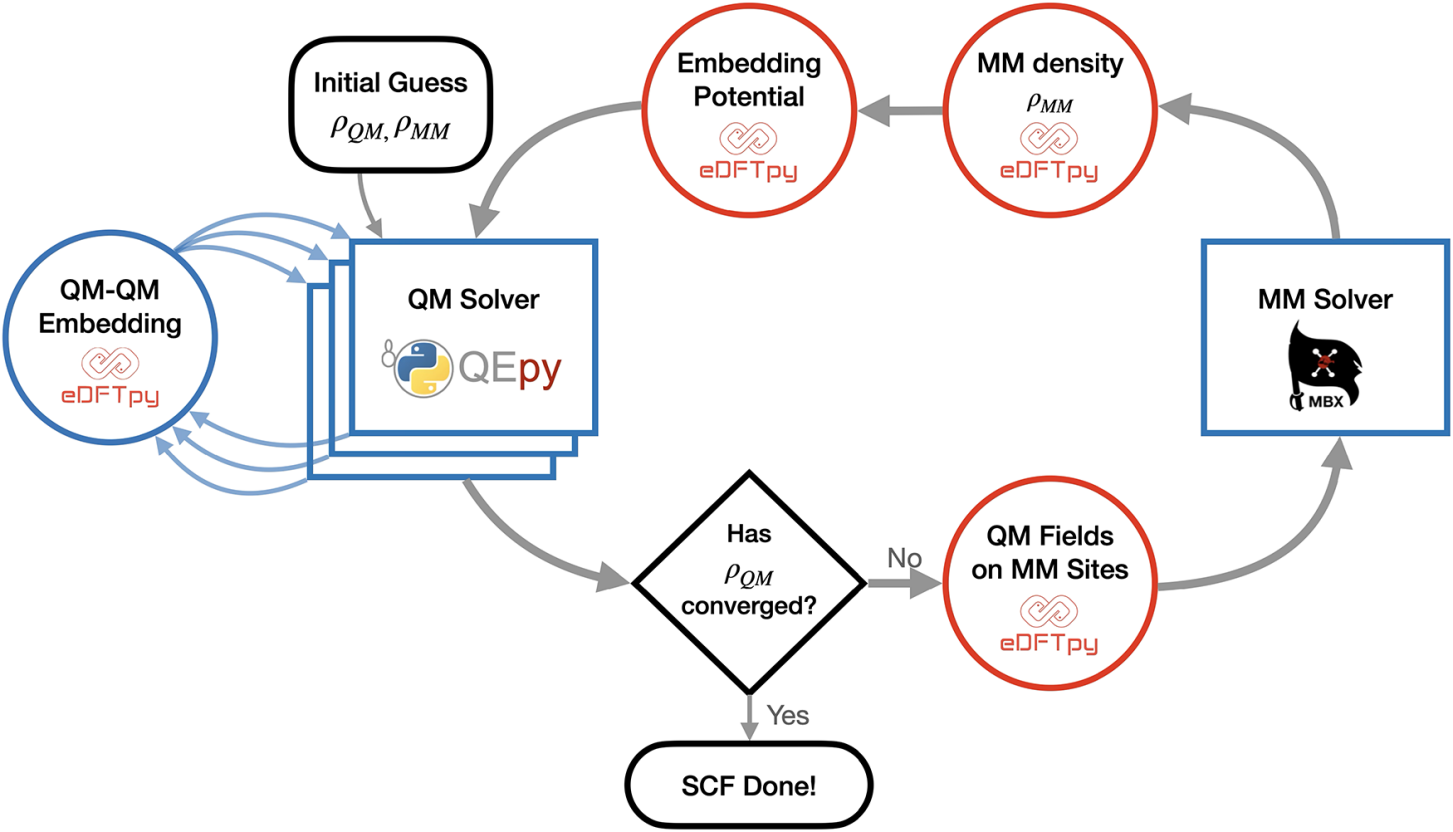
Workflow of the “density functionalized” QM/MM method with emphasis on software implementation. See details in section 3.

eDFTpy then instructs QEpy, the QM solver, to calculate the electronic structure of the QM subsystem. This is done using a single QEpy instance when only one QM subsystem is present, or multiple QEpy instances when several QM subsystems are included. For systems with more than one QM region, eDFTpy is responsible for computing the QM-in-QM embedding potentials, as indicated by the blue arrows connecting the QEpy solvers to the eDFTpy module in Figure 1. The QM electrostatic field, formally defined as the negative gradient of the MM embedding potential in eq 8, −∇⃗v_emb_^MM^(**r**), is evaluated at the sites of the MM polarizable dipoles.

For large systems, representing the QM electric field at the MM sites can be computationally demanding.^33,34,90,91^ To address this, we represent the QM electric field at the real-space grid point nearest to each MM dipole site. This approach is sufficiently accurate due to the smoothness of the QM field in the MM region,^34^ and it avoids the need to interpolate the field to the exact positions of the ions, which would require a costly MPI_GATHER operation. In eDFTpy, the MM cell is covered by a real-space grid that is fine enough to accurately represent the ionic pseudopotentials at each QM ion and MM site. In our simulations, we use a grid with a 100 Ha cutoff, which corresponds to a real-space grid spacing of approximately 0.23 *a*_0_.

Once the electric field is represented on the MM sites, the MM solver is tasked with solving the Coulomb problem in the MM cell to yield the MM polarizable dipoles. The MM density is then built using eq 6. Having the MM density and the density of all QM subsystems allows eDFTpy to compute the required embedding potentials for each subsystem, including the MM subsystem. The SCF cycles then continue until convergence is achieved, which is calibrated on the convergence of the QM density. At convergence, QM density and MM dipoles are fully mutually polarized.

In the eDFTpy implementation we take full advantage of plane wave reduction techniques that were developed for sDFT.^75,76,92–94^ Specifically, MM and QM subsystems are represented by different simulation cells, grids and thus plane wave basis sets. The QM simulation cell is smaller compared to the physical cell which coincides with the MM cell. Coulomb fields and other long-ranged energy functionals are evaluated on the large, physical cell. Employing such a multicell/grid approach is crucial in the context of QM/MM simulations as the MM subsystem is usually dramatically more extended than any of the QM subsystems. In the Supporting Information^68^ we further discuss our parallelization strategy.

The current implementation also features analytic energy gradients for the atoms in the QM region for the “charge only” implementation (omitting the MM dipoles) and approximately also for the full polarizable QM/MM implementation. In the Supporting Information we devote a section to the implementation of the forces and results are shown in Tables S3 and S4.

## 4 Computational Details

### 4.1 Pseudopotentials, Density Functionals and Plane Wave Cutoffs

GBRV pseudopotentials^84^ are used for all elements considered of both the QM and MM subsystems. The PBE exchange-correlation functional^95^ and the revAPBEk kinetic energy functional^96^ are employed for approximating additive *E*_xc_ and nonadditive *E*_xc_ and *T*_s_ functionals. More nuanced nonadditive functionals could be considered, and they would likely require a functional-specific reparametrization of the MM Gaussian widths. A good design principle for the QM-MM interaction functional is to employ the most accurate nonadditive functionals available for the systems considered. As in this work we consider water-solvated systems, and as revAPBEk was shown to be exceptionally well-suited for this type of systems,^72–74^ we chose revAPBEk for all examples featured here. All calculations include Grimme’s D3 correction.^97^ We choose plane wave basis sets for the QM subsystems with a cutoff energy of 20 Ha for wave functions and 200 Ha for charge density and potential unless otherwise stated (see supporting Table S1). The energy convergence threshold for the SCF was set to 10^–8^ Ha/atom. The Brillouin zone was sampled by a 5 × 5 × 1 *k*-point mesh for MoS_2_ and the Γ point for all other calculations. MM calculations were conducted using the MBX package^89^ using the MB-PBE and MB-Pol water models, which were developed to quantitatively reproduce PBE or CCSD­(T) water,^83,98^ respectively.

All inputs/output files, Jupyter notebooks needed to analyze the data and reproduce tables and figures in this work, as well as links to tagged versions of the software (MBX, eDFTpy, QEpy, and DFTpy) used for the simulations, are available as reported in the Supporting Information.^68^

### 4.2 Parameters Defining the MM Density

As described in the sections above, the proposed framework relies on the conversion of classical permanent charges and polarizable dipole sites into a smooth electronic density. For each permanent charge and polarizable dipole, this involves the fit of the width, σ, of the corresponding normalized Gaussian functions that contribute to the expansion in eq 6. In our applications to solvated systems, MM sites only involve oxygen and hydrogen atoms, for a total of 4 parameters that need to be fitted.

We also considered an additional MM-induced dipole self-energy correction. Although the induced point-dipole self-energy is given by $\frac{1}{2}\mu^{2}\alpha^{-1}$ ,^99^ where α is the isotropic dipole polarizability, we recognize that the dipoles considered in our work are not point dipoles, e.g., their charge density overlaps with the QM density at the QM/MM interface. Thus, we added an additional term to the self-energy for each site equal to *k*_*i*_^SE^|μ⃗ – μ⃗′|^2^, where μ⃗′ is the induced dipole at the same site when only the MM subsystem is considered, and *k*_*i*_^SE^ are element-dependent proportionality constants. Additional details about this correction and about how the permanent charge density was generated for the MB-PBE and MB-Pol force fields (which use the off-atom center M-site) are available in the Supporting Information document.^68^

The parameters defining the MM density were fitted so as to reproduce the QM-QM interaction energies for a single water molecule in bulk water. Ten snapshots of 64-water molecule cubic systems were taken from ref 100. These provided 640 water-bulk interaction energies. More details about this system will be given in the Section 5. The final values of the parameters for both MB-PBE and MB-Pol force fields are listed in Table S1.

The use of bulk simulations as a reference for the parametrization of the density functionalization approach provides a robust and general strategy that reduces the need for application-specific benchmarks. Results on small water clusters and solvation effects on molecules and materials (reported in the following) highlight the transferability of the obtained parameters.

## 5 Results and Discussion

### 5.1 Water Dimer

We start by comparing the QM/MM potential energy curves shown in Figure 2 and the polarization density depicted in Figure 3 for water dimers. The dimer structures, sourced from ref 101, were placed in a 20 Å cubic simulation cell and evaluated against benchmark QM/QM simulations performed using sDFT.

**Figure 2:**
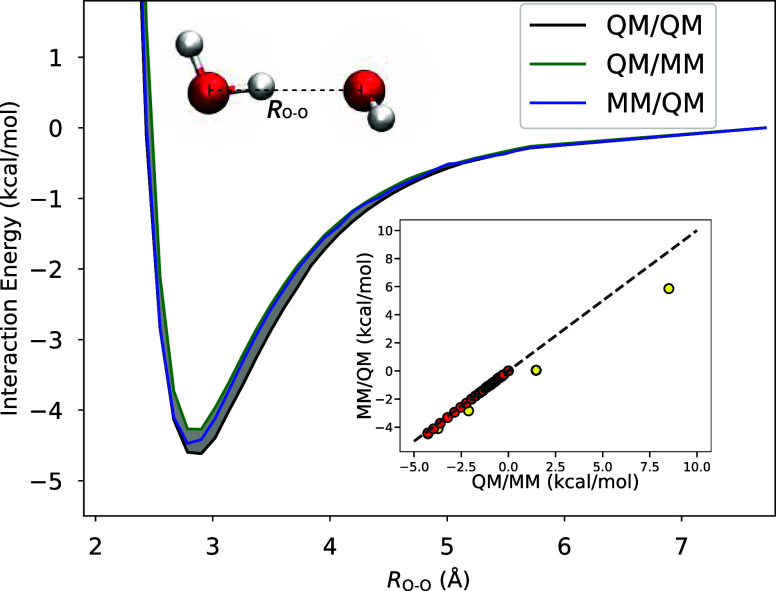
Water dimer energy curve (structure shown) for O–O distances ranging from 2.3 to 7.7 Å. The black line represents the QM/QM result, where both the donor and acceptor monomers are treated at the QM level. The shaded area represents the deviation of the QM/MM result from QM/QM. Green and blue lines correspond to the QM hydrogen bond donor (QM/MM) and acceptor (MM/QM), respectively. The inset shows a correlation plot of QM/MM and MM/QM interaction energies. Yellow markers represent geometries in the repulsive region of the curve (*R*_O–O_ < 2.9 Å), while red markers correspond to those in the attractive region (*R*_O–O_ > 2.9 Å).

**Figure 3:**
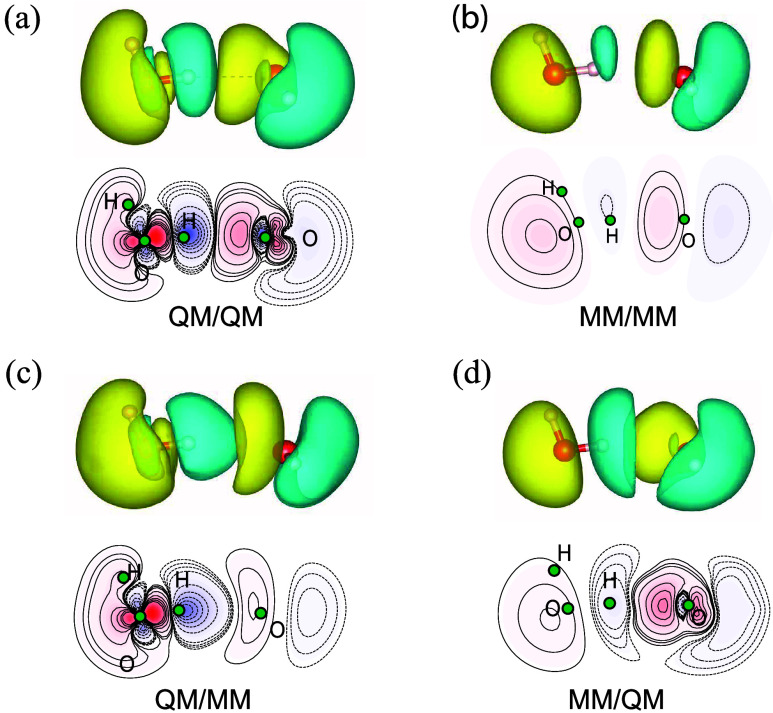
Polarization density, defined as ρ­(**r**) – ρ_iso_(**r**), where ρ_iso_ is the sum of the electron densities of the isolated water monomers, for the water dimer at the equilibrium O–O distance. In each panel, we present isosurfaces (top) and contour plots (bottom) generated with a cutoff of ±0.0007 *e*·*a*_0_^–3^. (a): reference QM/QM calculation; (b): MM/MM calculation carried out with MB-PBE, the dipoles have been represented by smooth Gaussians; (c) QM/MM calculation where the MM water molecule is the hydrogen bond acceptor; (d) MM/QM calculation where the MM water molecule is the hydrogen bond donor.

The key aspect of this system lies in its asymmetry, as one monomer is a hydrogen bond donor, and the other is an acceptor. Even though both QM and MM monomers are modeled by PBE (the MM subsystem is described by the MB-PBE model), the nature of the QM-MM interface is dramatically different whether one considers a QM hydrogen bond donor (QM/MM) or acceptor (MM/QM). Despite this, the curves are remarkably similar. The QM/MM and MM/QM minima (at 2.90 Å O–O distance) are less than 0.15 kcal/mol away from each other, and both deviate from the reference by a similar measure. These results show that the QM/MM interface is extremely well characterized by the nonadditive functionals. Crucially, the repulsive part of the curve is also well reproduced.

Our error of approximately 0.2 kcal/mol compares favorably to the 0.6 kcal/mol error reported in ref 43. for the dimer using polarizable QM/MM (MM treated with AMOEBA). We also observe about a 1 kcal/mol deviation when using another QM/MM (AMOEBA) implementation^22^ in our own calculations, as shown in Figure S1. These results demonstrate that a density functional treatment of the QM/MM interaction provides consistent interaction energies for both QM/MM and MM/QM partitions, whether the donor is treated at the QM level and the acceptor at the MM level, or vice versa. Previous studies have addressed inconsistencies between these two cases by introducing corrective short-range polynomials.^8^ In contrast, our formalism achieves this consistency directly, without requiring additional corrections. For completeness, we also compared a charge-only version of our density functional approach. In this implementation, the MM polarizable dipoles are omitted entirely, and only the permanent charges are retained. Specifically, the MM subsystem is represented solely by its electron density and the corresponding pseudopotentials, with no contribution from induced dipoles or polarization effects. We compared this charge-only scheme with the TIP4P model implemented in GPAW (see Figure S1). Although GPAW correctly yields consistent QM/MM and MM/QM results, our method achieves better agreement with the QM/QM reference, consistent with the observations reported by the GPAW authors in their paper.^28^

The inset of Figure 2 presents a correlation plot between the interaction energies obtained for the QM/MM and MM/QM systems, demonstrating that the two are in very close agreement. In the attractive region of the potential energy curve, the interaction energies follow the expected trend, with the data points lying along the diagonal. The repulsive region (highlighted by yellow markers) exhibits a slight deviation from this ideal behavior. This suggests there is some asymmetry in how charge penetration effects are captured for H_QM_-O_MM_ compared to H_MM_-O_QM_. To confirm that our findings are not influenced by artifacts from the use of periodic boundary conditions, we repeated the calculation of the interaction energy minimum in a larger simulation cell with a lattice constant of *a* = 50 Å. The result changed by less than 10^–3^ kcal/mol, indicating that finite-cell-size effects are negligible.

While agreement in interaction energies is necessary, it does not guarantee that a model accurately captures the underlying electronic structure. For a more stringent assessment, it is also important to compare the electron densities, and in particular, the polarization density of the system.

Figure 3 presents the polarization density (see caption for the definition) for the water dimer system. The MM polarization reproduces the overall features of the QM polarization only at a qualitative level. A direct comparison of the QM/QM case (panel a) with the MM/MM case (panel b) reveals that the MM approach misses several important details near the ion cores and along the O–O axis. These differences are well documented and reflect known limitations in the electrostatic response of dipole-only polarizable force fields.^102,103^ Notably, the QM/MM treatment leads to an improved description of the polarization in both monomers. As shown in panels (c) and (d), the MM molecule in the QM/MM setup exhibits polarization features that are prominent in the QM response but absent or only weakly present in the MM/MM case. This demonstrates that our approach handles the QM-MM interface accurately not only in terms of interaction energies (as seen in Figure 2) but also by the more stringent test of mutual polarization between subsystems.

### 5.2 Water Hexamer

Hexamer water structures play a crucial role in quantum chemistry due to their complex hydrogen-bond topologies. Accurately predicting their relative energies is often seen as a benchmark for a model’s ability to represent water across its various stable phases.^104^ Here, following ref 8, we focus on the most stable hexamer, the prism hexamer, to examine the effect of the QM-MM boundary. In the prism hexamer all water monomers act as both hydrogen bond donors and acceptors, with either 1/2 or 2/1 accepted/donated hydrogen bonds.

The root-mean-square errors (RMSEs) of the computed interaction energies defined as the energy of the hexamer minus the energy of the 6 isolated water molecules are collected in Figures S2 and S3 for the MM treated with MB-PBE and MB-Pol, respectively. When multiple water molecules are treated at the QM level, the sDFT framework allows flexibility in how the QM waters are grouped: they can be combined into a single subsystem (Frag. 1 in the figure) or divided into separate subsystems (Frag. 2). For each partition type with *k* QM or MM water molecules, there are $\left(\begin{array}{l} 6\\ k \end{array}\right)$ possible members of the partition (i.e., ways to split the hexamer into QM and MM subsystems).

Ideally, all members of each partition should yield the same interaction energy. Therefore, a larger RMSE for the predicted interaction energy indicates a lower accuracy of the method. Recognizing that no practical fragmentation method is perfect, we find a relation between the RMSE for the *k*-th partition (σ_QM/MM_(*k*)) and the square root of the number of members in the partition^105^ $\left(\sqrt{\left(\begin{array}{l} 6\\ k \end{array}\right)}\right)$ . This relationship is confirmed by *R* ^2^ values of 0.99 for all QM/MM and fragmentation methods considered (see Figure S4). The slope of this linear relationship is proportional to the average error per QM/MM boundary in the partitions ( $\mathrm{error}=\mathrm{slope}\cdot \sqrt{\frac{\pi }{2}}$ ), yielding a predicted error per boundary ranging between 0.2 and 0.3 kcal/mol for the methods considered. This error is consistent with the results for water dimers and, as we will see in the next section, also aligns with the RMSEs for the pentamer clusters extracted from bulk liquid structures.

### 5.3 Bulk and First Solvation Shell Water Environment

To further assess our method, we examine the dipole moment of individual water molecules and the interaction energy between them and their environment in bulk liquid water. As a benchmark, we use sDFT, which provides accurate subsystem electron densities and has been validated for various embedded molecular species.^60,100,106,107^ The analysis is based on 10 snapshots from an ab initio molecular dynamics trajectory of 64 water molecules in a cubic cell with a lattice constant of 12.42 Å,^69,100^ with all configurations provided in the Supporting Information.^68^

We adopt the notation *n*/*m* to indicate simulations with *n* molecules at the QM level and *m* molecules at the MM level, always totaling 64 water molecules. In each case, we report the interaction of a single water molecule with its environment. For example, in the 1/63 system, one water molecule is described quantum mechanically and the remaining 63 are described by the MM model. The sDFT benchmarks are generated both by treating all 63 environment molecules as a single subsystem (Figure 4) and by dividing them into individual subsystems (Figure S5); both approaches yield similar trends.

**Figure 4:**
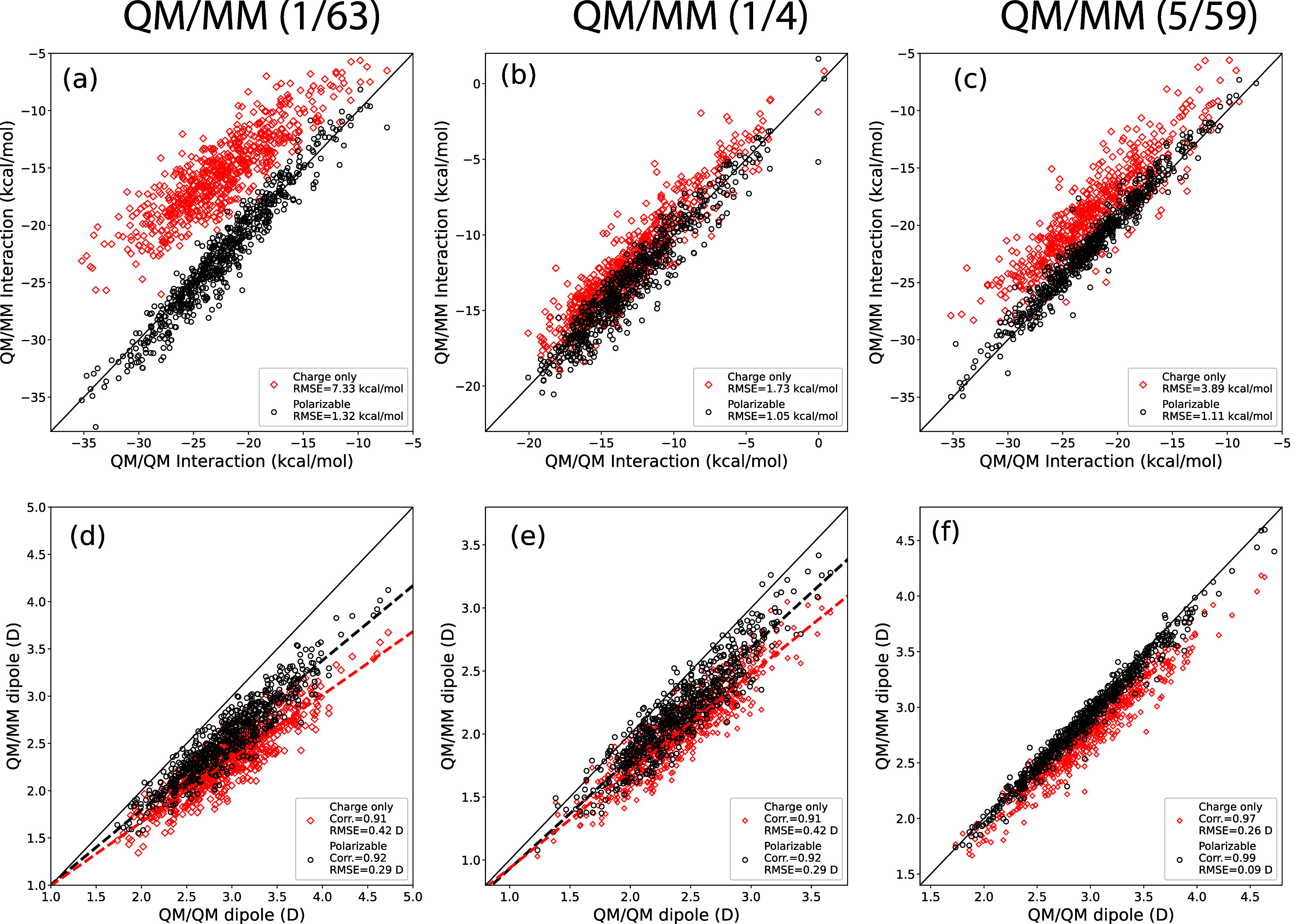
Panels (a)–(c): correlation plots of the interaction energy (in kcal/mol) of a single water molecule with its environment in a model of liquid water. (a) Bulk: QM/MM with 1 QM water molecule and 63 MM water molecules. (b) First shell: 1 QM water molecule and 4 MM water molecules (only the first solvation shell). (c) Minimal solvation: 5 QM water molecules and 59 MM water molecules. Panels (d)–(f): correlation plots of the dipole moment length of the embedded molecule (in Debye) for the same systems as for panels (a–c).

In addition to the bulk water configurations analyzed using the 1/63 partitioning, we examine two further cases inspired by ref 43. The first is the “first shell” configuration (1/4), in which each water molecule is surrounded only by its four nearest neighbors, resulting in a collection of isolated water pentamers rather than a bulk environment. The second is the “minimal solvation” setup (5/59), which is still representative of bulk water. Here, the target molecule and its four nearest neighbors are all treated at the QM level, with each water molecule modeled as a separate sDFT subsystem, while the remaining 59 molecules are treated at the MM level. For each scenario, we consider all water molecules in each of the 10 snapshots, resulting in 640 structures per case.

Panels (a) and (b) of Figure 4 show that the interaction energy between a water molecule and its environment in liquid water ranges from −35 to −7 kcal/mol in the bulk calculations, and from −20 to −1 kcal/mol in the first shell calculations. These energy ranges are consistent with values reported in the literature.^43^ As indicated in the figure, the root-mean-square errors (RMSEs) for the QM/MM interaction energies relative to the QM/QM benchmarks are 1.32 kcal/mol for the bulk system and 1.05 kcal/mol for the first shell. The minimal solvation setup shows an intermediate RMSE of 1.11 kcal/mol, improving on the bulk result. The RMSE for the first shell simulations is also comparable to the 1.4 kcal/mol reported for similar systems using the AMOEBA force field in ref 43. To our knowledge, there are no directly comparable results for the bulk or minimal solvation cases in the existing literature.

These RMSE values are also consistent with the accuracy achieved for the hexamer structure discussed earlier. Achieving an accuracy of about 1 kcal/mol for bulk systems is particularly notable, given that the average interaction energy per water molecule is significantly higher in bulk water (about 22.42 kcal/mol for QM–QM interactions) than in the hexamer (about 8.00 kcal/mol per water, using mbpol). This level of accuracy indicates that the mutual polarization between the QM and MM regions in both the bulk and minimal solvation setups is well captured by our QM/MM approach.

As illustrated in Figure 5, the polarization density of both the embedded water molecule (top panels) and the surrounding environment (bottom panels) is reproduced with reasonable fidelity in the QM/MM simulations. A noteworthy feature in the MM polarization of the environment is the relatively weak contribution from the second solvation shell compared to the QM/QM reference. This effect can be attributed to the use of slightly damped atomic dipole polarizabilities in typical polarizable force fields, as discussed in refs 108,109 Damping these polarizabilities is essential to avoid overpolarization, which in our context would otherwise arise from neglecting the nonadditive components of the embedding potential (see eq 8). For completeness, we show the polarization density from QM/MM with MB-pol in supporting figure S6. In future work, it will be valuable to examine how including these additional terms affects MM polarization and the choice of atomic dipole polarizabilities.

**Figure 5:**
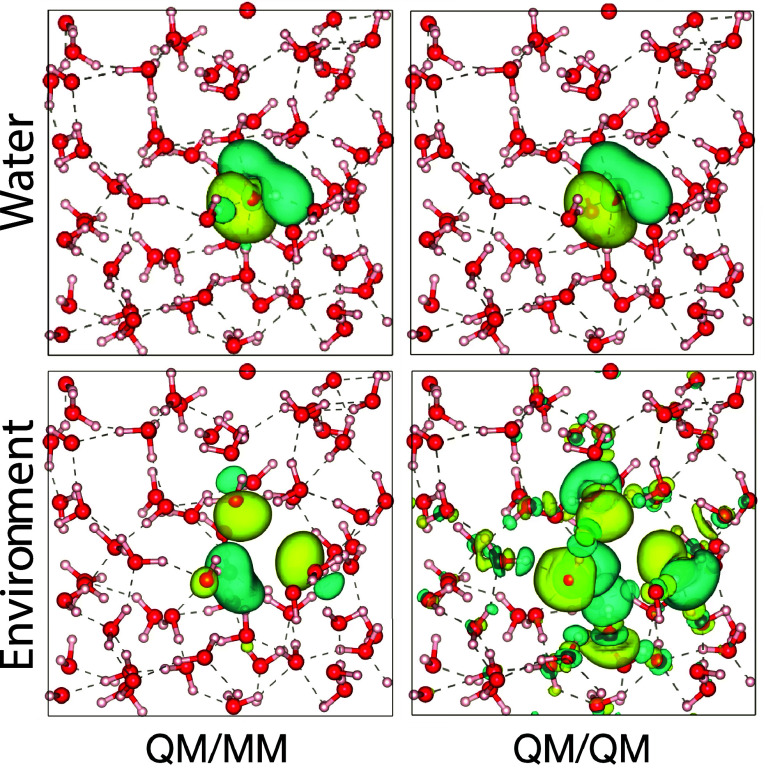
Polarization density (defined as the difference of the embedded and isolated molecular electron density) of an embedded water molecule employing the methods indicated in the figure. Top panels: single water molecule polarization. Bottom panels: environment polarization. The isosurface value is set to ±0.0025 e·*a*_0_^–3^.

Panels (d)–(f) of Figure 4 show the magnitude of the dipole moment for the central, embedded water molecule. The values range from 1.6 to 4.5 D in both the 1/63 and 5/59 bulk simulations, and from 1.3 to 3.5 D in the 1/4 first-shell calculations. Overall, the dipole moments are somewhat underestimated in the QM/MM simulations compared to the QM/QM reference. Despite this, the QM/MM and QM/QM (sDFT) dipole moments remain strongly correlated, with Pearson’s correlation coefficients above 0.9. For the 1/4 calculations, we find a root-mean-square error (RMSE) of 0.29 D, which compares favorably to the 0.62 D reported in ref 43. Together with the interaction energy results discussed above, these findings support our claim that our method achieves the highest accuracy for QM/MM simulations of liquid water reported to date.

Figure 4 also includes results for the charge-only implementation (red diamonds), in which the polarization of the MM subsystem is neglected by setting the polarizable dipoles to zero. For the first shell systems (1/4), the charge-only approach still yields reasonable results, with a dipole correlation of 0.91, a dipole RMSE of 0.42 D, and an interaction energy RMSE of 1.73 kcal/mol. However, for the bulk system, this approach performs poorly, as indicated by a significant increase in the interaction energy RMSE to 7.33 kcal/mol. This level of error is similar to what is typically observed in standard electrostatic embedding QM/MM simulations of condensed phases.^110,111^

Additionally, since MB-Pol provides a highly accurate benchmark for interaction energies, we include a comparison of our QM/MM interaction energies for the 1/63 system with MB-Pol reference values in supporting Figure S7.

### 5.4 Convergence with Respect to the QM Size

An important test for QM/MM simulations of solvated species is the convergence with respect to the number of water molecules included in the QM subsystem. As mentioned in the Section 1, having an accurate model for the QM-MM interface and employing an MM force field that is consistent with the QM method (we use the MB-PBE force field) should allow us to showcase strong QM/MM convergence. The typical target is a ±2 kcal/mol from the reference QM calculation.^42^ In Figure 6, we present the interaction energies of glucose (left panel) and the [Pd­(H_2_O)_4_]^2+^ aqua ion, counterbalanced by 2 Cl^–^ ions (right panel), with the water bulk environment as a function of the number of water molecules included in the QM subsystem. For glucose (a neutral molecule), the figure clearly shows that the ±2 kcal/mol target is reached (in fact, a ±1 kcal/mol is achieved) already when only 14 water molecules are included in the QM subsystem. This amounts to including less than the first solvation shell. Interestingly, the convergence for this system is much worse when a charge-only MM model is used (i.e., as done before, we simply neglect the polarization of the MM subsystem) for which 26 QM water molecules are needed to reach convergence. For PdCl_2_, despite the presence of a doubly charged cation, we find an essentially identical behavior with convergence being reached already with 13 QM water molecules. Conversely, the charge only QM/MM method does not reach the ±2 kcal/mol goal even when 46 QM water molecules are included. Therefore, we conclude that similar to the glucose system, including polarization in the MM subsystem dramatically improves the convergence of the interaction energy with the size of the QM subsystem.

**Figure 6:**
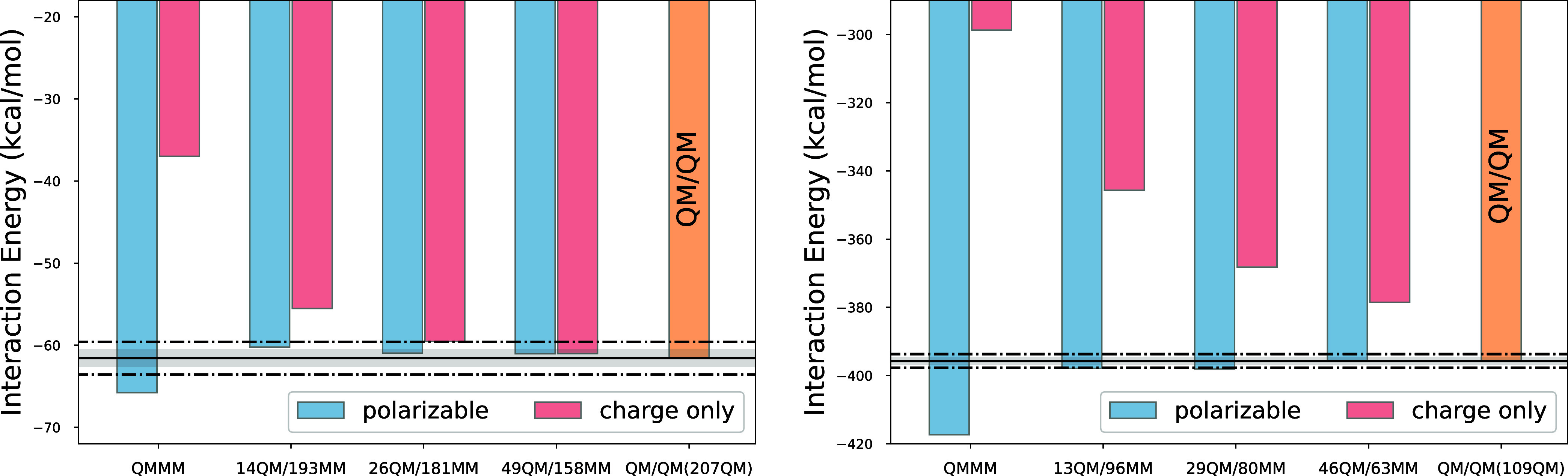
Convergence of the glucose (left panel) and the [Pd­(H_2_O)_4_]^2+^ aqua ion (counterbalanced by 2 Cl^–^ ions, right panel) solute-water interaction energy with respect to the number of water molecules included in the QM subsystem. An orange bar representing the sDFT reference is labeled as QM/QM in each plot. The dot-dashed line marks ±2 kcal/mol, and the shade marks ±1 kcal/mol window from the sDFT reference.

Our results on the need to include polarization in the MM subsystem find justification in the fact that, for a condensed phase system, including the inductive response of the environment, is crucial to obtain a physical picture, particularly for charged solutes.^43^

In supporting Figure S8, we also present the polarization density of glucose and its water environment, showing once again that the polarization of the QM/MM model qualitatively reproduces the polarization of the reference QM/QM simulation.

### 5.5 Charge Spill-Out Effects

As discussed in the Section 1, properly accounting for Pauli repulsion between the QM and MM subsystems is essential to prevent the well-known charge spill-out effect in the QM region. Our method addresses this by including nonadditive functionals, particularly the nonadditive kinetic energy functional, which introduces a repulsive term in both the energy and the potential felt by the QM electrons (see the term $\frac{\delta T_{s}^{\mathrm{nad}}}{\delta \rho_{\mathrm{QM}}\left(r\right)}$ in eq 7).

To illustrate the impact of this repulsive potential, we performed tests on the solvated PdCl_2_ system, comparing simulations with and without the inclusion of nonadditive functionals and their corresponding potentials (see Figure S9). The difference is clear: when the nonadditive terms are omitted, significant charge spill-out occurs from the Cl anions. This effect is mainly due to the absence of Pauli repulsion and is further exacerbated by the use of the PBE exchange-correlation functional, which, because of self-interaction error, causes Cl anions in vacuum to have partially unbound electrons and by the spatially delocalized nature of the basis set (plane waves). In the condensed phase, this problem is largely mitigated.^112^

We also show an example of wet MoS_2_ surfaces in the Supporting Information document.

## 6 Conclusion

We introduced a novel QM/MM framework that incorporates density-functional theory (DFT) for the QM and the MM subsystems that leverages an orbital-free treatment for the MM region and the QM-MM interaction. By assigning an electron density to the MM subsystem and accurately capturing nonadditive interactions (such as exchange, correlation, Coulomb, and Pauli repulsion effects) our density-functionalized QM/MM approach achieves chemical accuracy in modeling the interactions between complex solute–solvent systems. We validated the approach against a variety of water-based systems, including water clusters, bulk water, solvated ions, and a wet monolayer of MoS_2_, demonstrating consistent accuracy and achieving unprecedented fast convergence to chemical accuracy with respect to increasing size of the QM subsystem. Reaching chemical accuracy with the proposed method requires only the inclusion of the first solvation shell in the QM region–a significant advancement over traditional QM/MM schemes.

Particularly striking is the performance of our density functionalized QM/MM method for H_2_O in water. There we find that the excellent performance of the method for clusters (dimers, pentamers and hexamers) seamlessly translates to accurate models of bulk liquid water. A prime example is given by the dipole of the solute molecule which we predict an RMSE with QM/MM of 0.29 D, or 12%, for both bulk and pentamers compared to 0.624 D for pentamers from ref 43.

Our results highlight the critical role of (1) employing ab initio density functionals for the QM-MM interactions instead of ad-hoc parametrizations; (2) properly accounting for mutual polarization at the QM-MM interface; and (3) employing MM force fields that are consistent with the QM method employed (here we use MB-PBE for MM and DFT with the PBE exchange-correlation functional for QM). We found that following these three principles significantly improves convergence with respect to the size of the QM region compared to standard QM/MM methods. Our pilot simulations have showed that for both neutral and charged solutes, the interaction energies reached the target accuracy of within ±2 kcal/mol using a minimal QM region, with further refinement yielding sub-1 kcal/mol errors when merely the first shell of solvent molecules is included in the QM subsystem. This level of accuracy, achieved even in bulk water systems, underscores the robustness of our method for simulating condensed-phase environments.

Overall, this work presents a significant step forward in extending the applicability of QM/MM methods to treat larger, more complex systems than typically approached by standard QM methods while maintaining chemical accuracy. Future work will explore the impact of including beyond-Coulomb, ab initio terms in the MM embedding potential, so that the MM dipole response can more closely resemble the true electronic response of the MM subsystem. We also plan to apply the density-functionalized QM/MM framework to chemical environments other than water, for example those provided by biomolecules and materials interfaces as well as nonaqueous solvents.

## Supplementary Material



## Notes

Additional tables and figures and additional information related to (1) the definition of the QM-MM interaction functional; (2) the evaluation of atomic forces; (3) eDFTpy’s parallelization strategy, and (4) a calculation of wet MoS_2_ surfaces (PDF)
