# Supplementary material for: Density-Functionalized QM/MM Delivers Chemical Accuracy For Solvated Systems
Source: J Chem Theory Comput. 2025 Oct 15;21(20):10340–52. doi: 10.1021/acs.jctc.5c01440 (PMC12573752; doi:10.1021/acs.jctc.5c01440)
Supplement: Supplementary file 1 [file ct5c01440_si_001.pdf]

Supplementary Information Document for Density-Functionalized QM/MM Delivers Chemical Accuracy For Solvated Systems

Xin Chen,[†,‡,⊥] Jessica A. Martinez B.,[†,‡,⊥] Xuecheng Shao,[†,‡,¶,⊥] Marc Riera Riambau,[§] Oliviero Andreussi,[‖] Francesco Paesani,[§] and Michele Pavanello[*,†,‡]

†*Department of Physics, Rutgers University, Newark, NJ 07102*

‡*Department of Chemistry, Rutgers University, Newark, NJ 07102*

¶*Key Laboratory of Material Simulation Methods and Software of Ministry of Education, College of Physics, Jilin University, Changchun 130012, China*

§*Department of Chemistry, University of California-San Diego, San Diego, CA 92093*

‖*Department of Chemistry, Boise State University, Boise, ID 83725*

⊥*Authors contributed equally to this work.*

E-mail: m.pavanello@rutgers.edu

Additional Tables

Table S1: Parameter sets defining the MM electron density used in this work. Gaussian widths are expressed in Bohr (a_0). *Due to the presence of the M-site in both force fields considered, an additional density of permanent charges was used for evaluating the non-additive xc and kinetic energy functionals having $\sigma_q(\mathrm{H}) = 0.305$ and $\sigma_q(\mathrm{O}) = 0.850$ and $\sigma_q(\mathrm{H}) = 0.41$ and $\sigma_q(\mathrm{O}) = 0.742$ for MB-PBE and MB-Pol, respectively. See section of this document.

MM force field	$\sigma_q(\mathrm{M})^*$	$\sigma_q(\mathrm{H})^*$	$\sigma_\mu(\mathrm{O})$	$\sigma_\mu(\mathrm{H})$	k_i^{SE} $(\mathrm{Ha} \cdot a_0^{-2})$
MB-PBE	0.850	0.305	1.60	1.60	7.5
MB-Pol	0.742	0.410	1.77	1.37	18.5

Table S2: Summary of the simulation cells and data (plane-wave grids) employed in this work. MPI Gather operations between KS and QM+MM system are handled by region communicators (see text for details).

**System**	**Cell / large or small**	**Grid / ecut for $\rho(\mathbf{r})$**	**MPI Communicator**
KS subsystems	Subsystem / small	Subsystem / 200 Ha	`COMM_SUB`
MM subsystem	Supersystem / large	no grid	`COMM_SUB`
QM+MM system	Supersystem / large	Supersystem / 100 Ha	`MPI_COMM_WORLD`

Table S3: Analytic vs. numerical (finite-difference) forces for atoms in the QM subsystem, including different step sizes and SCF thresholds. Here, we consider one H-atom of the HB donor water molecule in the water dimer system presented in the main text. Finite differences forces are calculated with the five-point stencil formula.

SCF threshold	Step size (Å)	Numerical Force (Ha/Å)
10^{-4}	0.001	0.034499
	0.005	0.035729
	0.01	0.035193
	Analytical	0.034688
10^{-5}	0.001	0.034626
	0.005	0.034696
	0.01	0.034623
	Analytical	0.034689
10^{-6}	0.001	0.034726
	0.005	0.034716
	0.01	0.034700
	Analytical	0.034689
10^{-7}	0.001	0.034687
	0.005	0.034687
	0.01	0.034689
	Analytical	0.034689

Table S4: RMSE and total Forces in Ry/a_0 for Glucose embedded in 207 water molecules using QM/MM (MB-PBE) and QM/QM (sDFT). RMSE are taken with respect to sDFT optimized geometry.

	**RMSE (Å)**		**Forces (Ry/a_0)**	
**Optimization Point**	**QM/MM**	**QM/QM**	**QM/MM**	**QM/QM**
0	0.35	0.35	38	38
10	0.25	0.25	0.086	0.10
20	0.21	0.21	0.025	0.026
30	0.16	0.17	0.017	0.016
40	0.12	0.096	0.0080	0.013
50	0.11	0.063	0.0058	0.0095
60	0.098	0.049	0.0043	0.0061
70	0.088	0.038	0.0037	0.0045
80	0.082	0.026	0.0026	0.0033
90	0.081	0.020	0.0023	0.0027
Final	0.077	0.015	0.0011	0.00093

Additional Figures

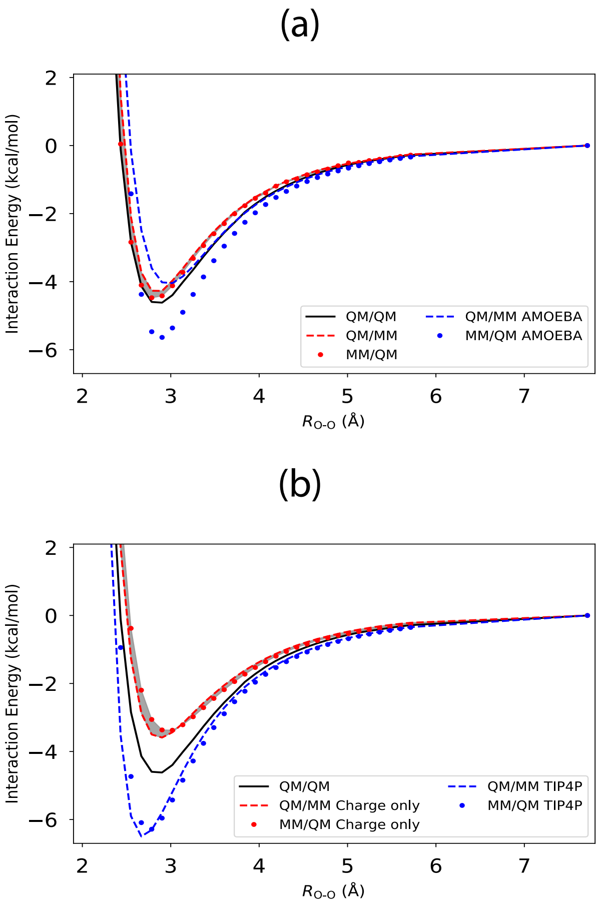

Figure S1: Comparison with other QM/MM methods. Potential energy curve of the water dimer (same system considered in the main text). We use the same notation as in the main text. (a) QM/MM and MM/QM indicate our method. AMOEBA indicates a calculation carried out with PySCF/OpenMMPol[1] using the 6-31G* basis set for the QM water and the AMOEBA polarizable force field for the MM water. (b) Comparison of the charge only QM/MM model against the QM/MM implementation in GPAW with TIP4P force field for the MM water.[2] In all cases, the shaded area indicates the discrepancy between our method's QM/MM and MM/QM results.

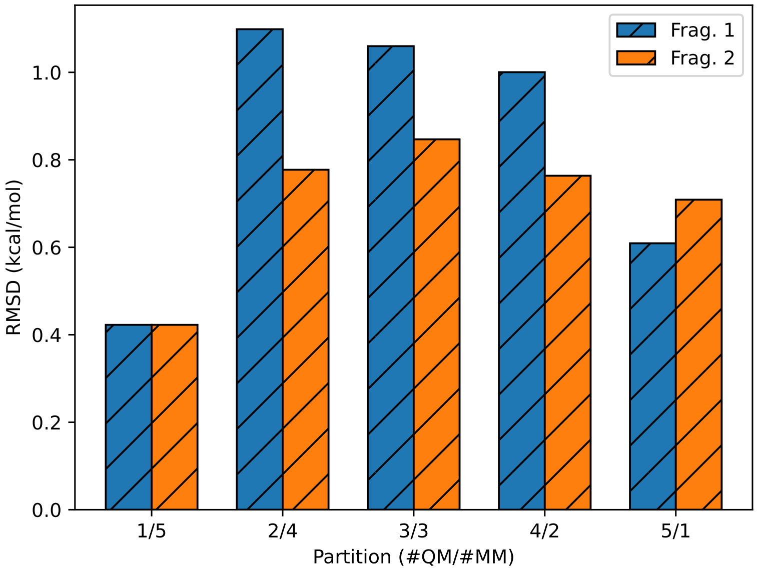

Figure S2: RMSEs of the total interaction energy (kcal/mol) of the prism water hexamer from QM/MM calculations versus the QM/MM partition type given by the number of QM and MM molecules in the calculations. Two fragmentation methods are considered (see main text). The MB-PBE MM solver was used.

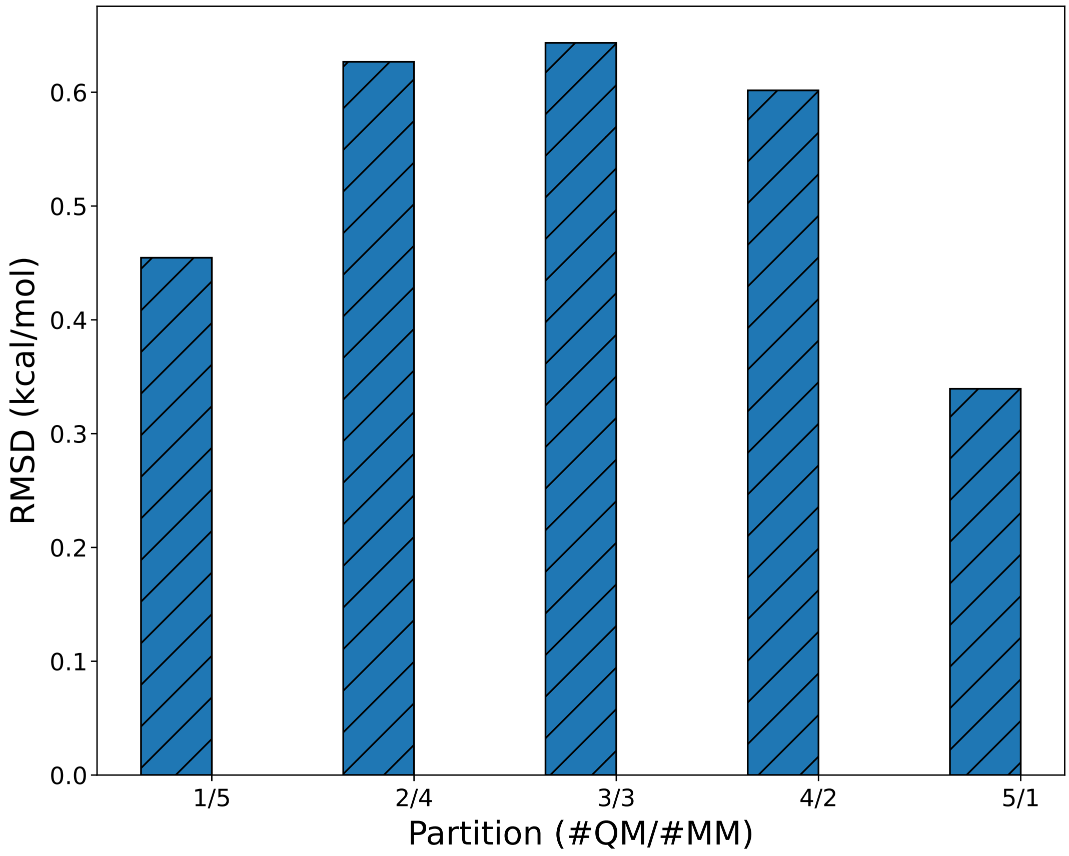

Figure S3: RMSEs of the total interaction energy (kcal/mol) of the prism water hexamer from QM/MM calculations versus the QM/MM partition type given by the number of QM and MM molecules in the calculations. Fragmentation type 2 was considered. The MB-Pol MM solver was used.

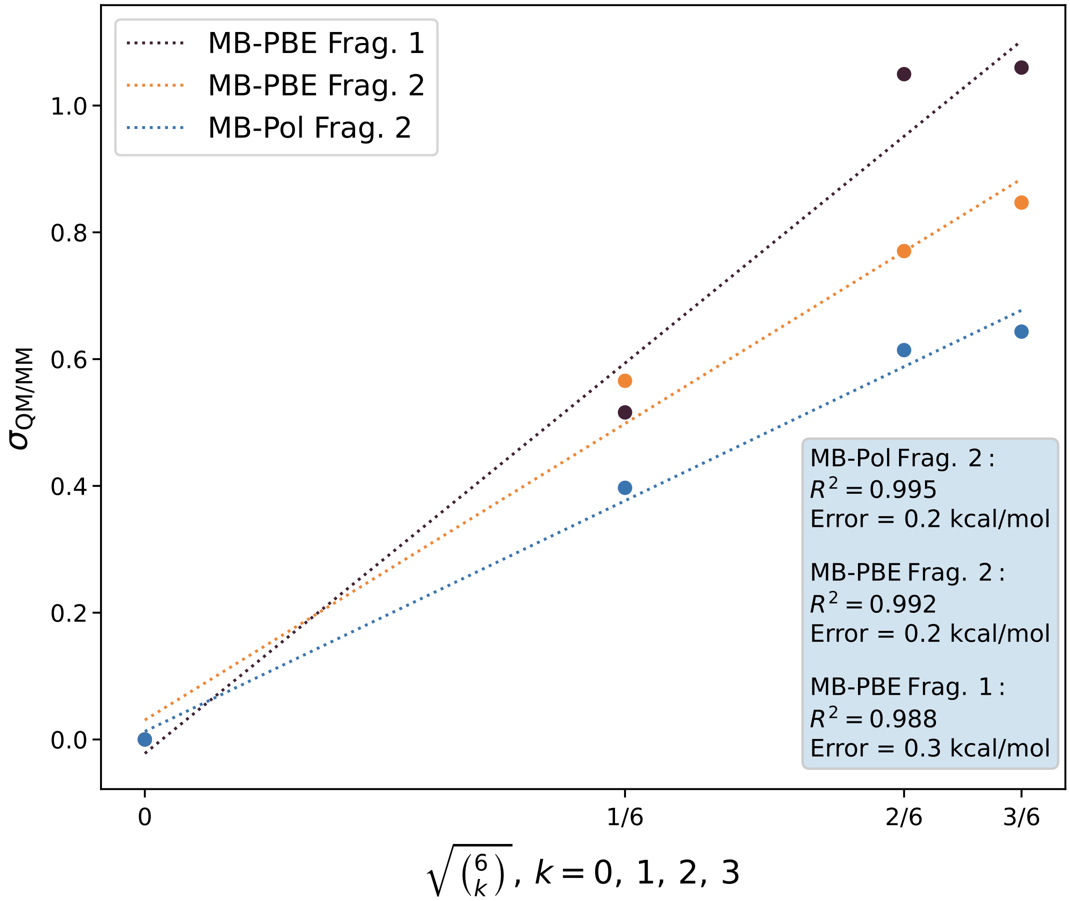

Figure S4: RMSD vs the square root of the number of water molecules in the minority set (QM or MM) of the prism water hexamer, labeled on the x-axis as the number of molecules in the minority set / total number of molecules.

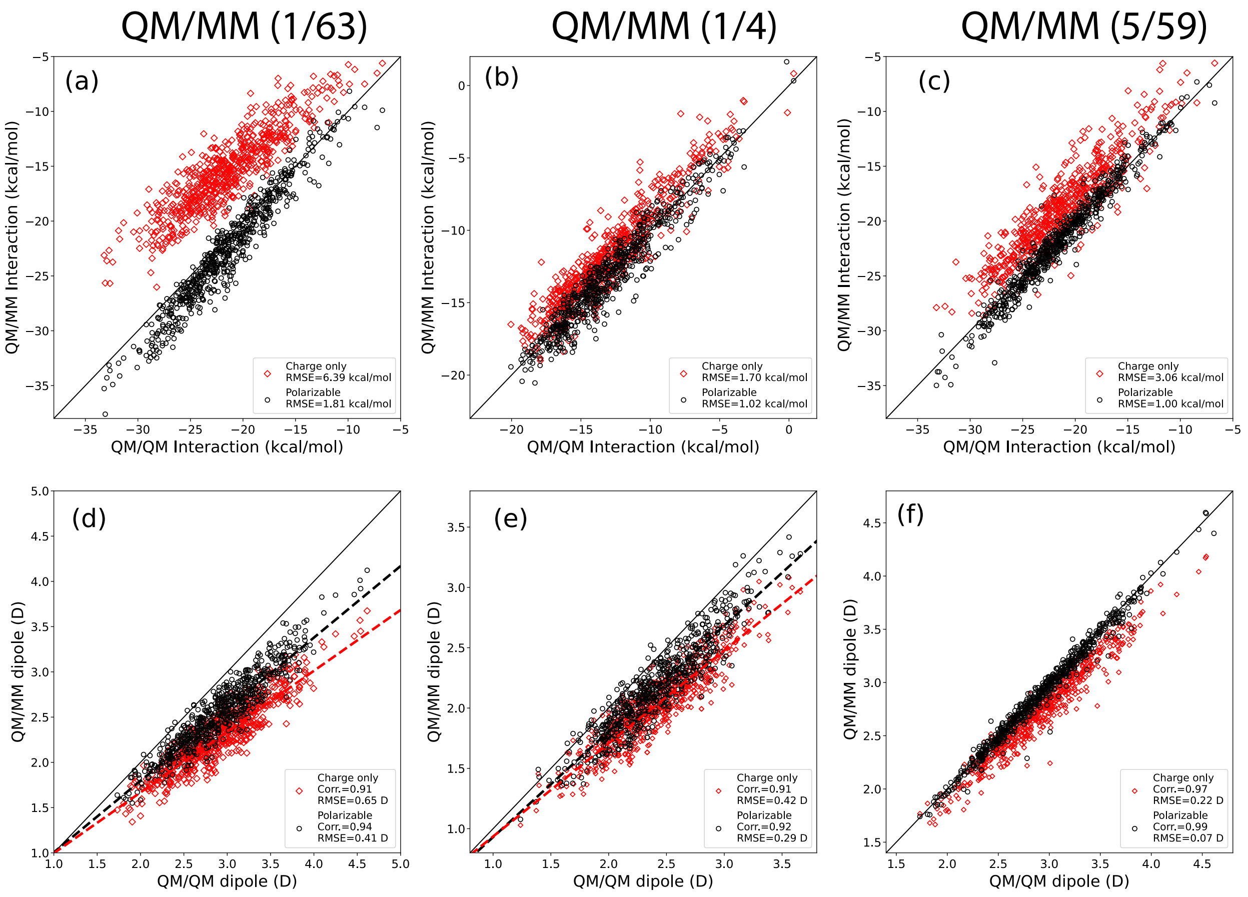

Figure S5: Panels (a), (b) and (c): interaction energy correlation plots (in kcal/mol) for the interaction of a single water molecule with either bulk (panels (a) and (c)) or first solvation shell (panel (b)) water environment. (a) QM/MM with 1 QM water and 63 MM water molecules. (b) 1 QM water and 4 MM water molecules (only the first solvation shell). (c) 5 QM waters and 59 MM waters. Panels (d), (e), and (f): correlation plots of the dipole moment length (in Debye) for the same systems as for panels (a–c). The MB-PBE force field for the MM subsystem is employed throughout. The benchmark QM/QM method used here for the x-axes differs from the one presented in the main text. In this analysis, we employ an sDFT setup with 64 coupled subsystems per calculation (one water molecule per subsystem) whereas the main text describes a setup with only 2 subsystems: a 1-water subsystem interacting with a 63-water subsystem.

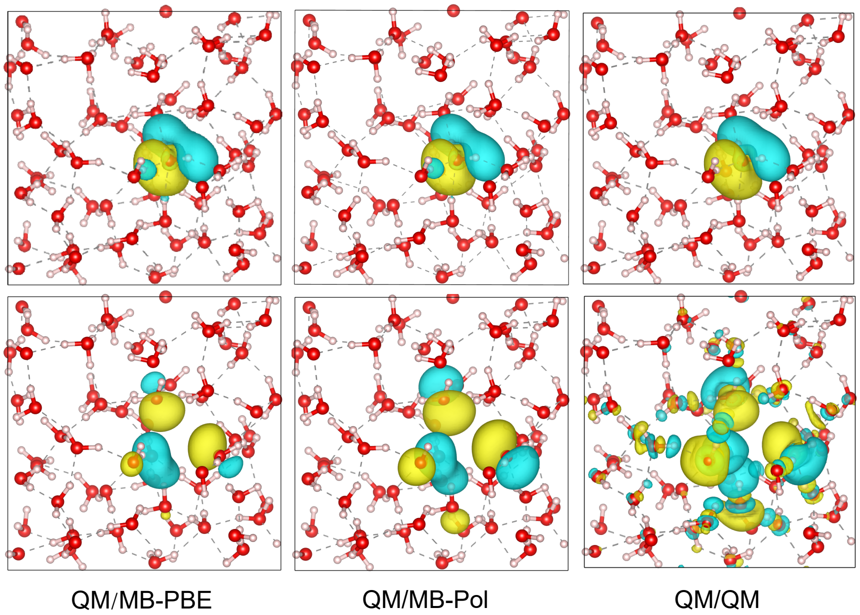

Figure S6: Polarization density (defined as the difference of the embedded and isolated molecular electron density) of an embedded water molecule employing the methods indicated in the figure. Top panels: single water molecule polarization. Bottom panels: environment polarization. The isosurface value is set to $\pm 0.0025\ e/a_0^3$.

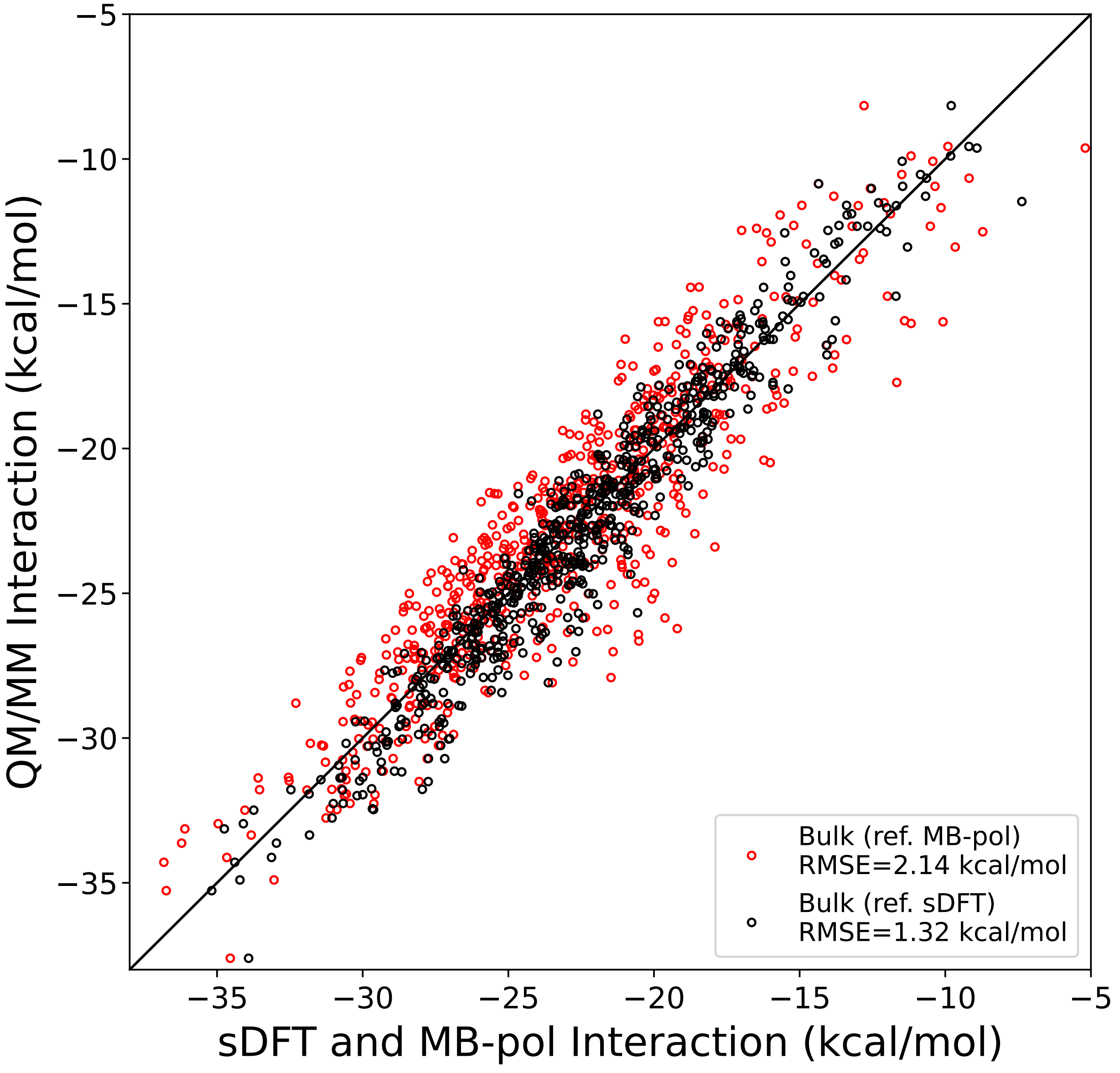

Figure S7: Correlation plots of interaction energies (in kcal/mol) for a single water molecule interacting with the bulk. The comparison includes QM/MM with one QM water molecule embedded in 63 MM water molecules (using the MB-PBE force field) against the MB-pol force field (red circles), and sDFT (with one QM water molecule embedded in a subsystem of 63 water molecules, black circles).

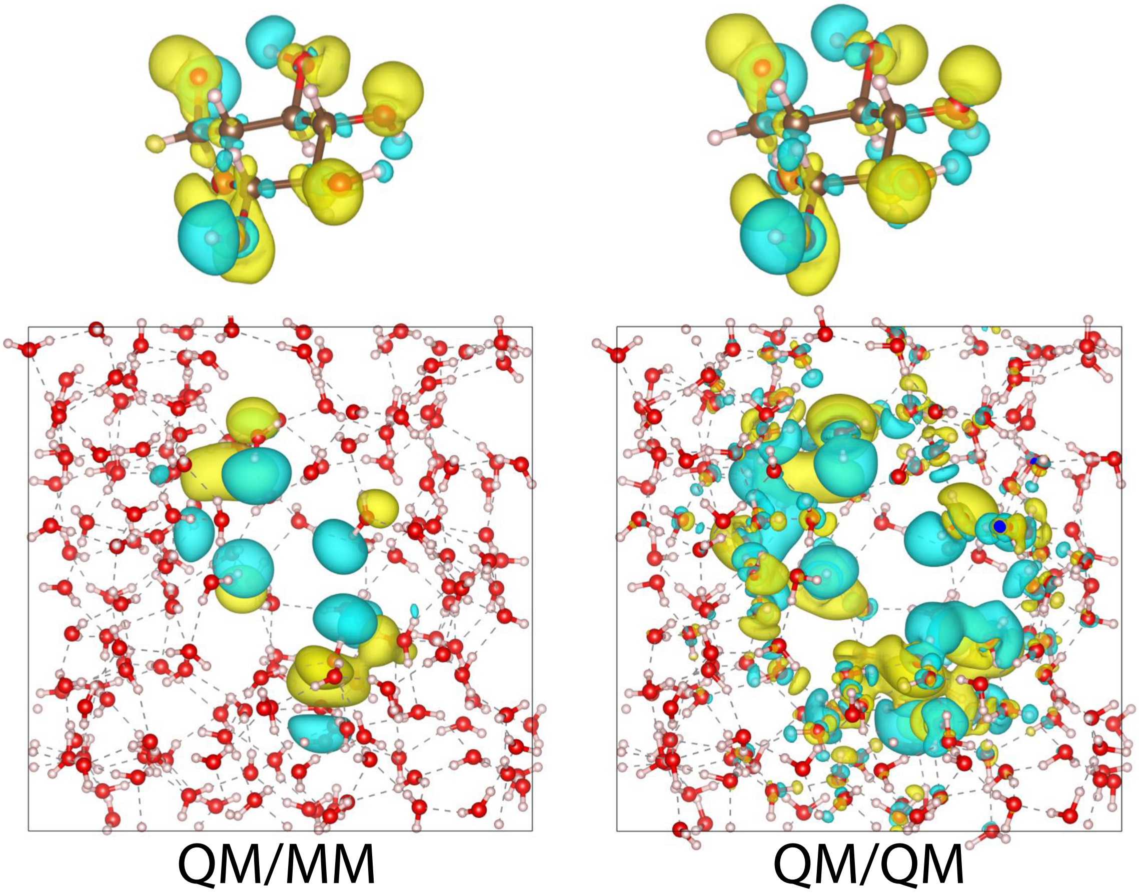

Figure S8: Polarization density (defined as the difference between the embedded and isolated molecular electron density) of a system composed by one glucose molecule and 207 water molecules. Right QM-QM (sDFT). Left QM-MM (MB-PBE). The isosurface value is set to $\pm 0.0025\ e/a_0^3$.

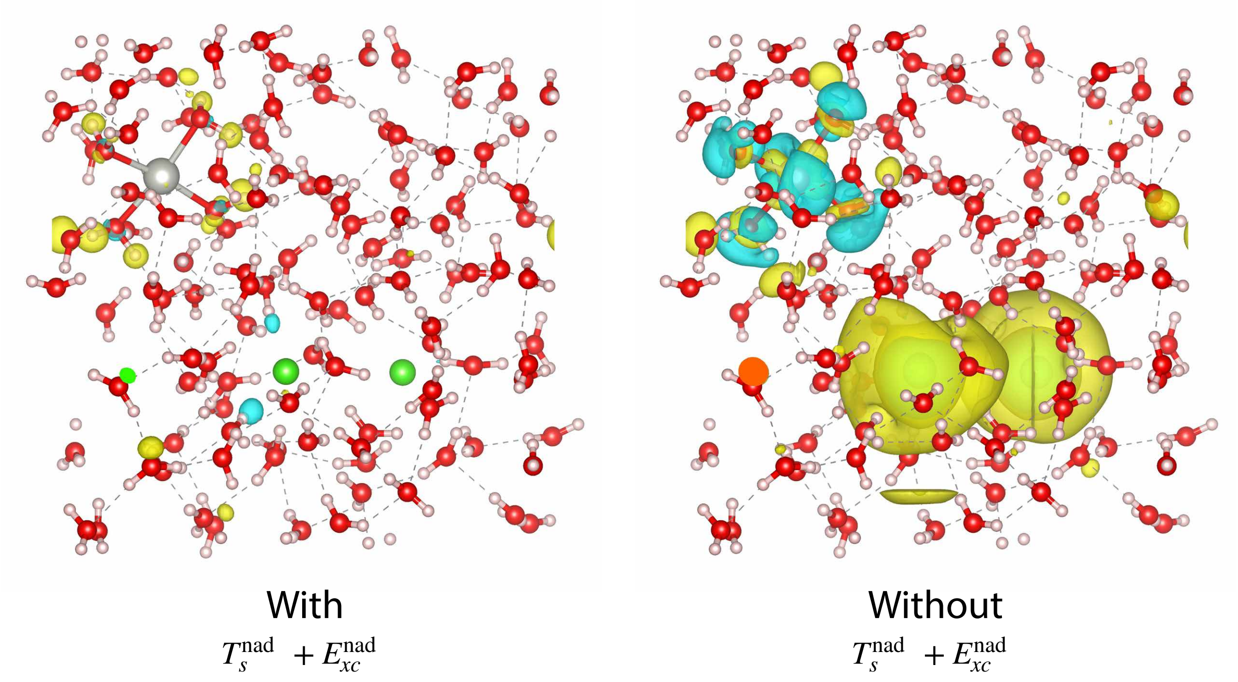

Figure S9: Effect of the non-additive exchange-correlation and kinetic energy functionals on the electron density of the QM subsystems for aqueous $PdCl_2$ (Pd ion in the upper left, Cl anions in the lower right of the simulation cell). The isosurfaces (isosurface value of $\pm 0.02\, e/a_0^3$) depict $\Delta\rho(\mathbf{r}) = \rho_{QM/MM}(\mathbf{r}) - \rho_{QM/QM}(\mathbf{r})$ that is the difference of the converged electron density of the QM/MM calculation with the density of the QM/QM calculation. Absence of nonadditive functionals causes the Cl subsystems to "spill out" electron density into the water bulk.

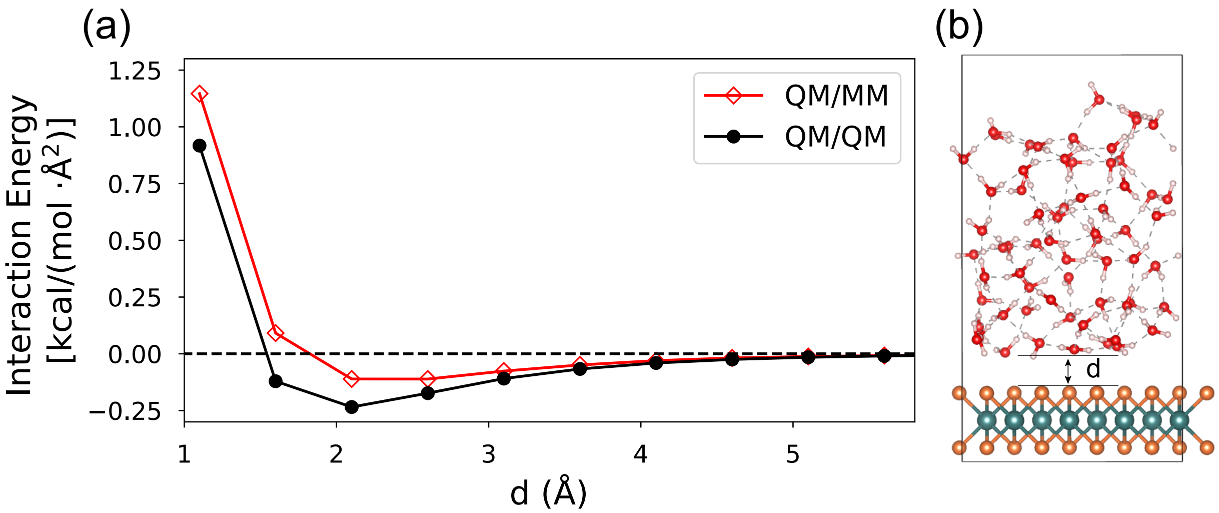

Figure S10: (a) Comparison of interaction energies between bulk water and MoS_2 at varying distances, with the red line representing sDFT total energy and the black line from QM/MM calculation. The distance is defined in panel (b) as the separation between the bottom of the water layer and the top of the MoS_2 surface.

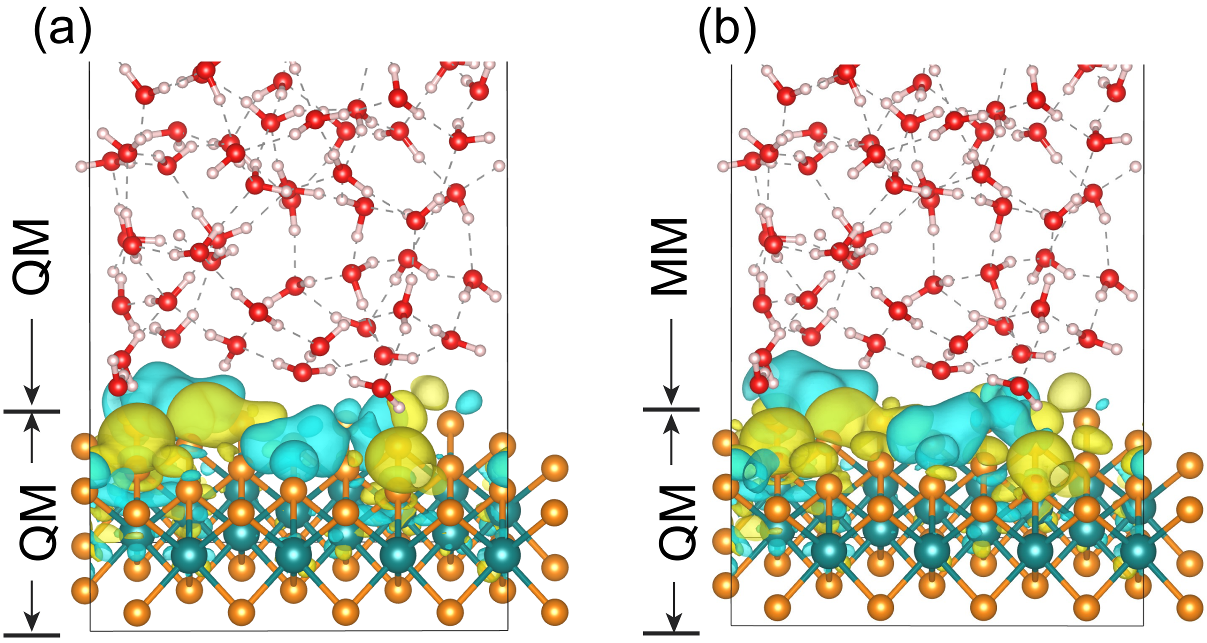

Figure S11: Polarization density plots illustrating MoS_2 polarization induced by water. (a) QM/QM reference (sDFT). (b) QM/MM calculation, where all water molecules belong to the MM subsystem. Isosurface level is $\pm 0.0030\, e/a_0^3$

QM/MM interaction energy

Induced dipole self-energy correction

The total energy of the molecular mechanics (MM) part, E_{MM}, consists of the energy contribution from the MM force field (MB-PBE and MB-Pol computed by MBX), E_{MBX}, as well as an additional contribution from the induced polarization self-energy δE_{MM},

$$E_{MM} = E_{\mathrm{MBX}} + \delta E_{MM} \tag{S1}$$

Such additional contribution accounts for the energy needed to generate the polarization in the MM subsystem due to its interactions with the QM subsystem. The MM dipoles are treated as point dipoles within the MM solver (MBX) but are seen by the QM system as a smeared charge density (see Eq.(5) of the main text). This discrepancy calls for a correction of the self-energy of the induced dipoles in the MM part by the QM subsystem.

To derive such a correction, we first consider the polarization density $\delta\rho$ of the MM subsystem due to its interactions with the QM subsystem. A Taylor expansion of the total MM energy functional reads as follows,

$$\begin{aligned} \delta E_{MM} &= E_{MM}\left[\rho + \delta\rho\right] - E\left[\rho\right] \\ &\cong \int \left.\frac{\delta E_{MM}}{\delta\rho(\mathbf{r})}\right|_{\rho=\rho_{MM}} \delta\rho(\mathbf{r})d\mathbf{r} + \int \frac{1}{2}\left.\frac{\delta^2 E_{MM}}{\delta\rho(\mathbf{r})\delta\rho(\mathbf{r}')}\right|_{\rho=\rho_{MM}} \delta\rho(\mathbf{r})\delta\rho(\mathbf{r}')d\mathbf{r}d\mathbf{r}' + h.o. \end{aligned} \tag{S2}$$

Since the MM density is an artificial density for which the exact KS potential is unknown, we make the assumption that ρ_{MM} corresponds to the ground state of the MM subsystem and set the first term in the Taylor expansion to zero. Consequently, an approximation for δE_{MM} is derived, noticing that the second functional derivative is simply the sum of the T_s, E_H and E_{xc} functional derivatives (kernels)

$$\delta E_{MM} \approx \frac{1}{2} \int \left(f_{T_s}(\mathbf{r}, \mathbf{r}') + \frac{1}{|\mathbf{r} - \mathbf{r}'|} + f_{xc}(\mathbf{r}, \mathbf{r}') \right) \delta\rho(\mathbf{r}) \delta\rho(\mathbf{r}') d\mathbf{r} d\mathbf{r}' \approx \sum_i \kappa_i^{SE} \mu_i^2, \tag{S3}$$

where $\mu_i = |\vec{\mu}_i - \vec{\mu}_i'|$. Here $\vec{\mu}_i$ is the induced dipole at the i-th site and $\vec{\mu}_i'$ is the same when only the MM subsystem is considered. κ_i^{SE} are parameters.

Coulomb and nonadditive terms and densities

The interaction energy E_{int} between QM and MM subsystems is composed of two main components: the Coulomb interaction term $E_{\text{int}}^{\text{Coul}}$ and the nonadditive term $E_{\text{int}}^{\text{NAD}}$.

$$E_{\text{int}} = E_{\text{int}}^{\text{Coul}} + E_{\text{int}}^{\text{NAD}} \tag{S4}$$

The Coulomb interaction component is represented as follows,

$$E_{\text{int}}^{\text{Coul}} = E^{\text{Coul}}\left[\rho, v_{\text{ext}}\right] - E^{\text{Coul}}\left[\rho_{\text{MM}}, v_{\text{ext}}^{\text{MM}}\right] - E^{\text{Coul}}\left[\rho_{\text{QM}}, v_{\text{ext}}^{\text{QM}}\right] \tag{S5}$$

The Coulomb energy of a system or subsystem arises from the contributions of the Hartree energy (electron-electron interaction energy), the electron-external potential energy, and the Ewald energy (ion-ion interaction energy), given by,

$$E^{\text{Coul}}\left[\rho, v_{\text{ext}}\right] = E_{\text{H}}\left[\rho\right] + \int \rho\left(\mathbf{r}\right) v_{\text{ext}}\left(\mathbf{r}\right) d\mathbf{r} + E_{\text{Ewald}}\left[v_{\text{ext}}\right] \tag{S6}$$

The non-additive interaction energy $E_{\text{int}}^{\text{NAD}}$ is additionally composed of contributions from the kinetic energy and exchange-correlation parts.

$$\begin{aligned} E_{\text{int}}^{\text{NAD}} &= T_s^{\text{NAD}} + E_{xc}^{\text{NAD}} \\ &= T_s[\rho] - T_s\left[\rho_{\text{QM}}\right] - T_s\left[\rho_{\text{MM}}\right] \\ &+ E_{xc}[\rho] - E_{xc}\left[\rho_{\text{QM}}\right] - E_{xc}\left[\rho_{\text{MM}}\right] \end{aligned} \tag{S7}$$

The water molecules are represented by a four-point TIP4P water model in MBX package. In this model, dipoles are located on O and H atoms, while the charges are located on the M sites and H atoms. This model can describe Coulomb interaction accurately. However, the MM electron density on M sites is shifted compared to the H atom sites. To address this inconsistency, we proposed a double density model, one density is used to calculate the Coulomb interaction, named as $\rho_{\mathrm{MM}}^{Coul.}$. The second density, $\rho_{\mathrm{MM}}^{\mathrm{NAD}}$ is used to calculate the non-additive interaction energy $E_{\mathrm{int}}^{\mathrm{NAD}}$.

$$\rho_{\mathrm{water}}(\mathbf{r}) = \begin{cases} \rho_{\mathrm{M}}(\mathbf{r}) + \rho_{\mathrm{H}}(\mathbf{r}) + \rho_{\mathrm{H}}(\mathbf{r}), & \text{Density for electrostatic interaction.} \\ \rho_{\mathrm{O}}(\mathbf{r}) + \rho_{\mathrm{H}}(\mathbf{r}) + \rho_{\mathrm{H}}(\mathbf{r}), & \text{Density for nonadditive interaction.} \end{cases} \tag{S8}$$

Further details on eDFTpy's parallelization strategy

A short note on our parallelization strategy is appropriate. Each instance of the QM and MM solvers are provided with their own MPI communicators, `COMM_SUB`. In all of our simulations, we use N MPI tasks of which $N-1$ are distributed to the QM subsystems and 1 is used for the MM subsystem. For the system sizes considered in this work (tens to a few hundred water molecules) we found it advantageous to maximize the processing power for the QM subsystems. Should much larger MM subsystems be considered (see for example the systems considered in Ref. 3) a larger share of processors can be allocated for the MM solver. At that stage, memory-conserving modifications to the current handling of the MM electrostatics (especially for MM sites far away from the QM subsystems) will be required. In addition to the subsystem communicators, eDFTpy uses system communicators which are attached to a grid. For the global QM+MM system, we use the large, physical cell which is spanned by the grid mentioned in the paragraph above. Such grid is distributed over the `MPI_COMM_WORLD` communicator as it is the most extended grid in the simulation. The subsystem cells are discretized by the subsystem grids which are handled by `COMM_SUB`. The total QM+MM density, $\rho(\mathbf{r})$, is recovered by gathering region communicators `COMM_REGION`

which are associated with non-overlapping portions of the QM+MM grid. This strategy proved successful also when considering large orbital-free DFT subsystems in sDFT simulations.[4] Key to the operation is to avoid `MPI_GATHER` operations on the full QM+MM grid. We summarize the grids and cells used in this work in Table S2.

Analytic forces on the QM subsystem's atoms

The implementation of forces on the atoms in the QM subsystem is identical to that used in standard QM/QM (sDFT) calculations, as described in Ref. 5, when polarizable dipoles are not included (i.e., in the "charge only" case). To validate our implementation, we benchmarked the analytical forces against those obtained by finite differences using a five-point stencil with step sizes of 0.01 Å, 0.001 Å, and 0.005 Å for the water dimer system. Specifically, we considered one of the hydrogen atoms of the hydrogen bond donor (treated at the QM level, with the acceptor treated at the MM level). As shown in Table S3, the analytical QM forces are in excellent agreement with the finite difference results. This agreement improves further as the SCF convergence threshold is tightened and does not depend strongly on the chosen step size.

A common strategy in polarizable force fields is to exclude charge–charge and charge–dipole interactions among MM sites within the same water molecule, as this helps prevent convergence issues during the induction of dipoles.[6] However, this exclusion complicates the force implementation. In our tests, we found that while the forces in the charge only case are accurate and well converged, including polarizable dipoles results in deviations from finite difference values on the order of 10^{-4} Ha/Å. This level of accuracy is sufficient for geometry relaxations (see Table S4 in the next paragraph), but for stable Born–Oppenheimer molecular dynamics, only the charge only approach yields reliably accurate forces.

To show the ability to run geometry optimization with our polarizable QM/MM method, we perform a relaxation of glucose solvated in 207 water molecules. First, we run a QM/QM

relaxation over the entire system. In a second step, we took this optimized structure and perturbed the Oxygen and Carbon atoms' positions, displacing the structure by an overall RMSD of 0.35 Å. With fixed degrees of freedom of the water molecules, we proceeded to the optimization of the glucose molecule. After 100 optimization steps, see Table S4, the QM/QM solver achieves a minimum RMSD of 0.015 Å, and the QM/MM reaches a similar value of 0.077 Å. This is $\approx$ 0.06 Å away from the initial equilibrated structure.

MoS_2 in water

To further demonstrate the versatility of our QM/MM framework, we applied it to a periodic system involving a monolayer MoS_2 surface solvated by water (see supplementary Figures S10 and S11). This test is important because it shows that our approach is not limited to molecular systems but is also suitable for modeling extended materials and interfaces, such as those found in two-dimensional materials and heterogeneous environments.

We considered a monolayer of MoS_2 wetted by 64 water molecules. The physical system is placed in a $12.72 \times 11.02 \times 38.27$ Å^3 simulation cell (structure depicted in Figure S10). In the sDFT and QM/MM simulations, the MoS_2 is described by a subsystem cell of dimensions $12.72 \times 11.02 \times 10.40$ Å^3. Unlike previous glucose in bulk water example, MoS_2 surface does not form hydrogen bonds with the water molecules solvating the surface.

In the first validation, we tested the interaction between water and MoS_2 at different distances [defined as d in Figure S10 (b)] from 1.1 to 5.6 Å. The interaction curves of QM/MM and sDFT show a similar trend. Generally, we notice that the surface-water interaction is underestimated by QM/MM. However, the repulsion at short distances ($<$ 1.5 Å) is well described. Both QM/QM and QM/MM curves feature shallow minima with a value of -0.23 and -0.11 kcal/(mol·Å), respectively. The location of the minimum is 2.1 Åfor both QM/MM and QM/QM schemes. The polarization at the water-surface interface is an important physical phenomenon. We image it in Figure S11. Water as a polar solvent induces dipoles

locally on the MoS_2 surface. Once again, the QM/QM and QM/MM polarization plots are semiquantitatively similar.

The results for the wet MoS_2 surface are consistent with our earlier findings. QM/MM simulations reproduce the QM/QM interaction energy with reasonable accuracy. For example, the equilibrium interaction energy predicted by QM/MM is -0.11 kcal/mol·Å^{-2}, compared to the QM/QM reference value of -0.23 kcal/mol·Å^{-2}. The polarization densities obtained with QM/MM are also very close to those from the QM/QM benchmark. Both methods predict an equilibrium water–MoS_2 distance of 2.1 Å.

Links to software repositories

- eDFTpy: *qmmm-dev* branch of eDFTpy version 64f6752 at `https://github.com/Quantum-MultiScale/eDFTpy`
- QEPy: *master* branch of QEpy version 8f2dc53a at `https://github.com/Quantum-MultiScale/QEpy`
- DFTpy: *master* branch of DFTpy version 081f1f69 at `https://github.com/Quantum-MultiScale/DFTpy`
- MBX: *master* branch of MBX version 635950f at `https://github.com/paesanilab/MBX`

Links to Jupyter notebooks analyzing the raw outputs

The data (input/output) corresponding to this paper's calculations, figures, and analysis are available at the Zenodo repository under DOI: 10.5281/zenodo.16755573.

References

(1) Bondanza, M.; Nottoli, T.; Nottoli, M.; Cupellini, L.; Lipparini, F.; Mennucci, B. The OpenMMPol library for polarizable QM/MM calculations of properties and dynamics. *J. Chem. Phys.* **2024**, *160*.

(2) Dohn, A.; Jonsson, E. O.; Levi, G.; Mortensen, J. J.; Lopez-Acevedo, O.; Thygesen, K. S.; Jacobsen, K. W.; Ulstrup, J.; Henriksen, N. E.; Møller, K.; others Grid-based projector augmented wave (GPAW) implementation of quantum mechanics/molecular mechanics (QM/MM) electrostatic embedding and application to a solvated diplatinum complex. *J. Chem. Theory Comput.* **2017**, *13*, 6010–6022.

(3) Reinholdt, P.; Kongsted, J.; Lipparini, F. Fast approximate but accurate QM/MM interactions for polarizable embedding. *J. Chem. Theory Comput.* **2021**, *18*, 344–356.

(4) Shao, X.; Mi, W.; Pavanello, M. Density embedding method for nanoscale molecule–metal interfaces. *J. Phys. Chem. Lett.* **2022**, *13*, 7147–7154.

(5) Genova, A.; Ceresoli, D.; Pavanello, M. Avoiding fractional electrons in subsystem DFT based ab-initio molecular dynamics yields accurate models for liquid water and solvated OH radical. *J. Chem. Phys.* **2016**, *144*.

(6) Babin, V.; Leforestier, C.; Paesani, F. Development of a "first principles" water potential with flexible monomers: Dimer potential energy surface, VRT spectrum, and second virial coefficient. *J. Chem. Theory Comput.* **2013**, *9*, 5395–5403.
